# State‐of‐the‐Art Advancements in Photocatalytic Hydrogenation: Reaction Mechanism and Recent Progress in Metal‐Organic Framework (MOF)‐Based Catalysts

**DOI:** 10.1002/advs.202103361

**Published:** 2021-10-29

**Authors:** Mengya Guo, Mingwei Zhang, Runze Liu, Xiangwen Zhang, Guozhu Li

**Affiliations:** ^1^ Key Laboratory for Green Chemical Technology of Ministry of Education School of Chemical Engineering and Technology Tianjin University Tianjin 300072 China; ^2^ Collaborative Innovative Center of Chemical Science and Engineering (Tianjin) Tianjin 300072 China

**Keywords:** MOFs‐based photocatalyst, photocatalytic hydrogenation, reaction mechanism, unsaturated chemicals |CO_2_

## Abstract

Photocatalytic hydrogenation provides an effective alternative way for the synthesis of industrial chemicals to meet the economic and environment expectations. Especially, over the past few years, metal‐organic frameworks (MOFs), featured with tunable structure, porosity, and crystallinity, have been significantly developed as many high‐performance catalysts in the field of photocatalysis. In this review, the background and development of photocatalytic hydrogenation are systemically summarized. In particular, the comparison between photocatalysis and thermal catalysis, and the fundamental understanding of photohydrogenation, including reaction pathways, reducing species, regulation of selectivity, and critical parameters of light, are proposed. Moreover, this review highlights the advantages of MOFs‐based photocatalysts in the area of photohydrogenation. Typical effective strategies for modifying MOFs‐based composites to produce their advantages are concluded. The recent progress in the application of various types of MOFs‐based photocatalysts for photohydrogenation of unsaturated organic chemicals and carbon dioxide (CO_2_ ) is summarized and discussed in detail. Finally, a brief conclusion and personal perspective on current challenges and future developments of photocatalytic hydrogenation processes and MOFs‐based photocatalysts are also highlighted.

## Graphical abstract

In this review, the background and development of photohydrogenation are summarized. Photocatalysis is compared with thermal catalysis, and the fundamental understanding of photohydrogenation is concluded. Moreover, MOFs‐based photocatalysts, their typical modification strategies, and applications in photohydrogenation are discussed. Finally, the conclusion and perspective on challenges and developments of photohydrogenation and MOFs‐based photocatalysts are highlighted.

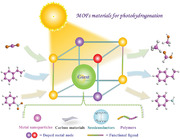

## 1 Introduction

Hydrogenation is among the central themes of petrochemical, coal chemical, fine chemical, and environmental industries.^[^ ^1^ ^]^ As one of the backbones of the chemical industry, it has a wide range of applications in the synthesis of various products.^[^ ^2^ ^]^ It is estimated that 25% of chemical transformations include at least one hydrogenation step. Hydrodenitrogenation and hydrodesulfurization processes are employed to remove N and S elements in crude oil.^[^ ^1^ ^]^ Selective hydrogenation is the direct route to eliminate the impurities of fine chemicals. Traditional hydrogenation reactions are generally accomplished through thermal‐based chemical processes using H_2_,^[^ ^3^ ^]^ or small molecules hydrogen donor like alcohol, formic acid or triethanolamine,^[^ ^4^ ^]^ or stoichiometric reducing agent like NaBH_4_. In the high‐temperature thermal hydrogenation reactions, the exhaustion of energy sources severely affects sustainable development.^[^ ^5^ ^]^ From the viewpoints of energy, environment, cost and safety, the development of catalytic hydrogenation reactions under mild conditions is highly desirable. Moreover, the search for renewable energy resources for hydrogenation is of great significance nowadays.^[^ ^6^ ^]^ Solar energy has received extensive attention due to its abundance, sustainability and nonpolution. Essential energy and environmental issues of hydrogenation can be addressed by photocatalysis using solar energy.^[^ ^7^ ^]^

**Scheme 1:**
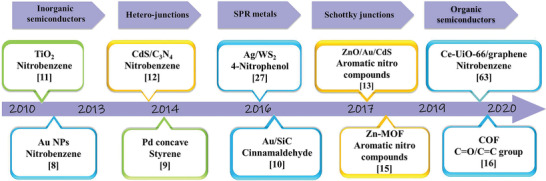
The timeline for the development of photohydrogenation reactions.

In the past decade, many photosensitive catalysts have been developed and applied to various photocatalytic hydrogenation reactions, and remarkable progress has been made as summarized in **Table** **1**. There have been only a few reviews of photolytic hydrogenation reaction, although much progress has been achieved. Systematic understandings of various photocatalytic hydrogenation mechanisms are urgently desired. **Scheme** **1** illustrates the key attempts and developments during the past decade in this field. Early in 2010, hot electrons were applied to organic hydrogenation by Zhu group.^[^ ^8^ ^]^ They creatively applied plasmonic Au nanoparticles (NPs) to drive the hydrogenation reaction of nitroaromatic chemicals. The inspiration for this enlightening work was originated from the following two points: first, Au NPs can be used as the active sites for nitrobenzene reduction; second, surface plasmon resonance (SPR) on Au NPs under visible light can further facilitate the reaction. SPR can generate hot electrons to heat Au NPs and activate the 6sp electrons of Au to a higher energy level. Then, the electrons are injected to molecular orbital of nitrobenzene, and thus assist the cleavage of the N—O bonds, which cannot occur in the dark.

**Table 1:** Typical photocatalysts for the photohydrogenation of organic chemicals

Reduced species	Catalyst	Substrate	Light source	Condition	Ref.
NaBH_4_	Ag/WS_2_	*p*‐Nitrophenol	Visible light	H_2_O	[27]
	Au/C_3_N_4_	Nitrophenols	Visible light	H_2_O	[28]
	Ag/TiO_2_	*p*‐Nitrophenol	Visible light	H_2_O	[29]
	Au/TiO_2_ or SiO_2_	4‐Nitrophenol	350–500 nm light	H_2_O	[30]
H_2_	Pd NCs	Styrene	Full spectrum	H_2_O	[9]
	Pd/Cu_2_O	Phenylacetylene	Blue LED	EtOH	[31]
	Cu@Zn/TiO_2_	quinolone	Visible light	toluene	[32]
	CoS_2_/graphene	Nitroaromatics	Visible light	EtOH	[33]
	Pd/SiC	Furan	Visible light	*n*‐Amyl alcohol	[34]
	AuAgPt	Phenylacetylene	Visible light	EtOH	[35]
	Au_6_–Pd_1_/SN	Cinnamaldehyde	Visible light	Cyclohexane	[36]
e^−^	TiO_2_	Nitro compounds	UV	Isopropanol	[11]
	PbBi_2_Nb_2_O_9_	4‐Nitroaniline	Visible light	H_2_O (NH_4_)_2_C_2_O_4_	[37]
	CdS/C_3_N_4_	Nitrobenzene	Visible light	Benzyl alcohol	[12]
	TiO_2_	Nitrobenzene	380–760 nm light	Benzyl alcohol	[38]
	DHN/TiO_2_	Nitrostyrene	Blue light	MeCN, TEOA	[39]
	Ag/C_3_N_4_	Nitrobenzene	Visible light	MeOH	[40]
	ZnO–Au@CdS	Aromatic nitro compounds	Visible light	H_2_O HCOONH_4_	[13]
	Pt–Au nanorod	Resazurin	Visible light	N_2_H_4_	[41]
H^•^ species	Pd/Si	Nitrobenzene	375–800 nm	2‐Propanol, HCOOH	[42]
	Pt/TiO_2_	Phenylacetylene	UV	CH_3_OH	[43]
	Pt/CN	C═C; C═O; N═O	420 nm	H_2_O 1,4‐dioxane TEOA	[44]
	PtPd alloy/TiO_2_	2‐Methyl‐3‐butyn‐2‐ol	UV	H_2_O CH_3_OH	[45]
	Pt/C_3_N_4_	5‐Hydroxymethylfurfural	Visible light	H_2_O TEA	[46]
e^−^ and H^•^ species	CoP@CdS	Nitroarenes	Visible light	H_2_O, HCOONH_4_	[47]
	Ni_2_P/CdS	Nitroarenes	Visible light	H_2_O, Na_2_S/Na_2_SO_3_	[48]
e^−^ and reducing free radical	CdS@MIL‐68	4‐Nitroaniline	Visible light	H_2_O, HCOONH_4_	[49]
	Au_1_Pt_2_/TN	Halonitrobenzenes	Visible light	CH_3_OH, HCOONH_4_	[50]
H^•^ species and reducing free radical	Pd/TiO_2_	Nitroaniline	Visible light	H_2_O, HCOONH_4_	[51]
	Pt/SWO‐NS	Nitrobenzene	Visible light	CH_3_OH, HCOONH_4_	[52]

^a)^ Notes: EtOH: ethanol; MeCN: acetonitrile; TEOA: triethanolamine; MeOH: methanol; TEA: triethylamine.

In 2015, Long group fabricated Pd concave nanostructures possessing plasmonic cross‐sections for directly harvesting light in a broad spectral range for styrene hydrogenation.^[^ ^9^ ^]^ This property thus induced higher solution temperature through photothermal effect to accelerate styrene hydrogenation. In addition, they discovered that the hot electrons generated on Pd made a negative contribution to styrene hydrogenation because of strong binding of hydrogen to Pd sites, which is opposite to the result on Au as reported by Zhu group.^[^ ^8^ ^]^ Although some plasmonic metals showed superior photocatalytic hydrogenation activity, their potential applications need to be further investigated. Guo and co‐workers constructed Mott–Schottky contact between Au and SiC for photocatalytic hydrogenation.^[^ ^10^ ^]^ In this system, the synergistic effect of LSPR (localized surface plasmon resonance) on Au NPs and the charge transfer across the Au/SiC interface contributed to high performance of cinnamaldehyde photocatalytic hydrogenation (TOF 487 h^−1^, 100% selectivity for alcohol). Additionally, nonplasmonic metals including Pd and Pt were also reported to be suitable for coupling with SiC to form Schottky junction, and both of them showed enhanced activity and selectivity.

Semiconductor photocatalysts have also been widely reported for photocatalytic nitroaromatic reduction. Several works employed conventional TiO_2_ in the selective reduction of various nitroaromatics to amines under UV light. Liu et al. fabricated TiO_2_ with controllable crystal forms and facets for photocatalytic reduction of nitrobenzene, in which the crystal‐structure‐dependent catalytic activities were revealed.^[^ ^11^ ^]^ Fabricating binary semiconductors heterojunctions with matched band structure could further facilitate the separation of photoexcited charge carriers, which promoted the reduction of reactant.^[^ ^12^ ^]^ Subsequently, Xu et al. introduced Au nanoparticles as the interfacial mediator into ZnO@CdS semiconductor heterojunction (type II) to form vectorial Z‐scheme ZnO–Au@CdS photocatalyst.^[^ ^13^ ^]^ This ternary heterostructure featured the Z‐scheme charge carrier transfer between ZnO and CdS. As a consequence, the electrons and holes with high redox ability were retained to efficiently participate in the photocatalytic redox reactions. For the reduction of aromatic nitro components, the photocatalytic activity of this unique system is 28 and 1.5 times higher than those of ZnO–Au and ZnO@CdS counterparts, respectively. Based on these pioneering works, many researchers turned their attention from traditional inorganic semiconductors to new organic porous photocatalysts.

In 2019, Qiu et al. constructed a series Zr‐based UiO metal‐organic frameworks (MOFs) through metal node substitution and ligand modification, and studied their photocatalytic performances systematically.^[^ ^14^ ^]^ This work shed a light on the fabrication of MOFs photocatalysts with high performance by molecular architecture modification. The easily tunable MOFs systems hold the great potential to become next‐generation heterogeneous photocatalysts for many challenging hydrogenation reactions. Chen and co‐workers presented the synthesis of a novel 3D visible‐light‐responsive Zn‐based MOF and its photocatalytic reduction application.^[^ ^15^ ^]^ The photogenerated electrons transferred from MOF to nitroaromatics enabled the selective reduction of nitroarenes to generate anilines. This work demonstrated the great potential of MOFs for the production of valuable chemicals via organic transformation. Recently, covalent triazine polymers (CTP), which famed for their structural diversity as well as tunable optical and electronic properties, have been used in photocatalytic H_2_ production and CO_2_ reduction. Hu and colleagues reported visible‐light‐photocatalytic hydrogenation of maleic acid and furfuryl on a metal‐free thiophene‐containing CTP with high production rate.^[^ ^16^ ^]^

MOFs, as a novel type of porous crystalline porous materials constructed from metal or metal clusters interconnected by organic ligands, featured with high specific surface areas, tunable pore structure,^[^ ^17^ ^]^ versatile structure design,^[^ ^18^ ^]^ have emerged as promising photocatalysts in the applications of organic degradation and transformation,^[^ ^19^ ^]^ water splitting^[^ ^20^ ^]^ and carbon dioxide (CO_2_) reduction.^[^ ^21^ ^]^ Because MOFs possess the following advantages. 1) Uniform and independent semiconductors are formed between each metal‐oxygen unit in MOFs, which is conducive to the separation of electrons and holes.^[^ ^14^ ^]^ 2) The high porosity of MOFs greatly suppresses the recombined volume of photoexcited electron and hole due to the shortened transport distance of charge carriers.^[^ ^7^ ^]^ 3) The cavities in the MOFs are suitable to host photoactive complexes as diverse catalytic centers. 4) MOFs can effectively improve the absorption and utilization efficiency of sunlight by screening different organic ligands and metal centers. However, some pristine MOFs still suffered from the disadvantages of poor ability of visible light adsorption, rapid recombination of photogenerated carriers, low electronic conductivity,^[^ ^22^ ^]^ limited pore space, and poor long‐term stability during photocatalysis including photostability, moisture stability and thermal stability. Up to date, many strategies have been employed to optimize the photocatalytic properties of MOF‐based composites.^[^ ^23^ ^]^ Based on the controllable and diverse structure construction, it is easy to fabricate MOFs with enhanced photocatalytic efficiency.

So far, great process has been achieved in the development of photohydrogenation over MOFs‐based photocatalysts. A majority of relevant reviews have been published to summarize the photocatalytic applications of MOFs in H_2_ production,^[^ ^23^, ^24^ ^]^ CO_2_ transformation^[^ ^5^, ^25^ ^]^ and organic degradation.^[^ ^26^ ^]^ However, the systematic summary respecting photocatalytic hydrogenation applications of MOFs‐based photocatalysts is still lacking. In this review, we will discuss state‐of‐the‐art photocatalytic hydrogenation reactions, whereby special attention is placed on the basic principles and mechanism as well as predominant factors affecting photocatalytic performance. Photocatalytic hydrogenation reactions of various substrates with different functional groups, including alkenes, aldehydes and functionalized nitroarenes will be focused. Herein, guidelines for the development of new photocatalytic hydrogenation systems are expected to be provided.

## 2 Photocatalysis versus Thermal Catalysis

In the field of thermal catalysis, the reactions are driven by the thermal energy following a thermal mechanism. Considerable efforts have been devoted to the proper design of the catalyst, including metal NPs (size, shape, and composition) and supports (surface property, defect, and pore structure), which can be used for reference in photocatalysis, especially photohydrogenation. With the development of photocatalysis, many reports have shown that suitable metal nanostructures and semiconductors in photocatalysts can effectively drive desired organic synthesis at moderate reaction conditions.^[^ ^53^ ^]^ Some works presented the differences between thermal catalysis and photocatalysis in hydrogenation process.^[^ ^25^, ^54^ ^]^ In this section, we will focus on the unique properties of photohydrogenation distinct from thermal catalysis in the following aspects: adsorption and activation of substrate, reaction pathway and kinetic mechanism modulation, various photohydrogenation pathways and selectivity regulation.

### 2.1 Adsorption and Activation of the Substrates

**Figure 1:**
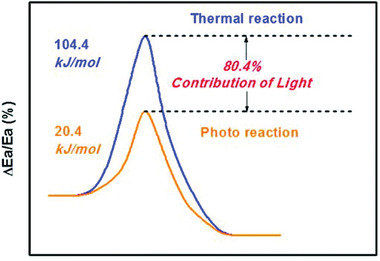
Apparent activation energies of nitrobenzene reduction calculated for the photoreaction and the thermal reaction in the dark. Reproduced with permission.^[^ ^56^ ^]^ Copyright 2016, Royal Society of Chemistry.

During the process of heterogeneous catalysis, the reactant molecules first adsorb on the catalyst surface,^[^ ^55^ ^]^ and then overcome the activation energy to form the product. In the field of thermally driven catalysis, the activation of reactant is originated from heat input. Photo energy from visible light can replace heat as another energy source to active reactant and drive the reaction. Liu et al. revealed that the apparent activation energy for the reduction of nitrobenzene under light condition was only 20.4 kJ mol^−1^, whereas it was 104.4 kJ mol^−1^ in the dark, as illustrated in **Figure** **1**.^[^ ^56^ ^]^ Under light irradiation, this catalytic hydrogenation process proceeded with reduced activation energy and higher nitrobenzene conversion rate. Many hydrogenation reactions are energy‐consuming processes which require high temperature to initiate. Encouragingly, under lower temperature, the reaction can be triggered thanks to the assistance of photoexcitation process. Moreover, adsorption mode and strength of the substrate are affected by electronic state of the active sites, which determines the product selectivity via a specific reaction pathway. The commonly used strategy to modify electronic properties of the active metal is engineering interaction between support and metal or introducing a second metal promoter. Nevertheless, it is still challenging to construct well‐defined structure with uniform active centers to recognize target functional group. Furthermore, the selectivity improvement is usually accompanied by a certain degree sacrifice of its activity. These problems are expected to be solved in the photocatalytic system. The separation and transfer of photogenerated electrons could further precisely regulate electronic state of the active sites. Accordingly, many photocatalysts offer enhanced reaction activity and selectivity under mild light illumination conditions compared to traditional catalysts.

### 2.2 Modulation of Reaction Pathway and Kinetic Mechanism

Experimental studies and theoretical calculations demonstrate that the photoexcited electrons make major contributions to the initiating and enhancement of reaction. Bimetal structures combining plasmonic metal with transition metal have attracted considerable attention, which are widely used in the field of photocatalytic organic transformation. Chen group chose Pt‐tipped Au nanorods (NRs) as a module catalyst for the reduction of resazurin by N_2_H_4_ to reveal catalytic kinetic mechanism on the bimetal structure containing plasmonic metal.^[^ ^41^ ^]^ Unexpectedly, the catalytic reactions on Au–Pt nanorods underwent totally different pathways with and without light illumination.

**Figure 2:**
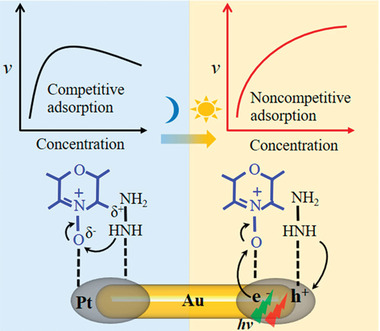
Reaction pathways for the reduction of resazurin by N_2_H_4_ on Au–Pt NRs before and after light irradiation. Reproduced with permission.^[^ ^41^ ^]^ Copyright 2020, American Chemical Society.

Competitive adsorption happened on the Au–Pt NRs in dark environment. Both reactants adsorbed competitively on the same type of active sites, leading to the suppression of catalytic activity at high concentration of reactants. Under light irradiation, the reaction pathway on the Au–Pt NRs followed a new noncompetitive‐adsorption model. A charge‐driven reaction route was proposed to account for this novel change: the catalytic active sites for converting resazurin and N_2_H_4_ were related to photogenerated electron sites and hole sites, respectively. N_2_H_4_ molecules acting as the sacrificial agent were trapped by holes, meanwhile, resazurin was reduced by photoinduced electrons. Obviously, this photocatalytic mechanism avoided the decrease in activity at high reactant concentration caused by competitive adsorption, which provides new idea for improving catalytic efficiency. In addition, the formation rate of photoinduced charge carriers on Pt caps can be boosted by nearby Au, and thus the reaction rates showed a dependence on irradiation intensity (**Figure** **2**).

### 2.3 Fundamental Understanding of Photocatalytic Hydrogenation

#### 2.3.1 Plasmonic Metal Nanoparticles Photocatalytic Systems

**Figure 3:**
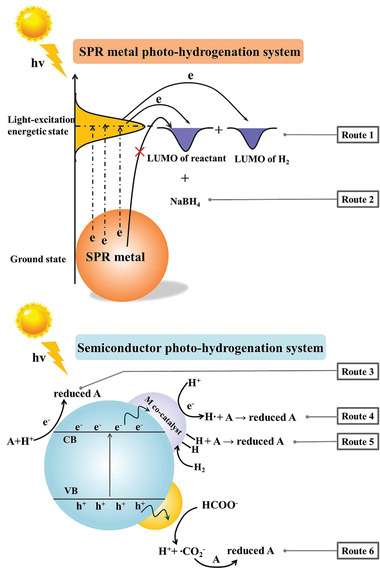
The pathways of photocatalytic hydrogenation reactions. LUMO: lowest unoccupied molecular orbital; M: metal.

For plasmonic metal photocatalytic system, H_2_ and NaBH_4_ were commonly used as the reducing agent and hydrogen source. **Figure** **3** summarizes the overall reaction mechanism of photocatalytic hydrogenation through six possible pathways. Routes 1 and 2 are the hydrogenation pathways commonly occurring over plasmonic metal photocatalysts. On plasmonic metal NPs, under the irradiation of light, the photoexcited electrons are excited to higher energy level to active the adsorbed substrate and/or H_2_, followed by the formation of intermediate active species to realize the hydrogenation reaction.

##### H_2_ as the Hydrogen Source and Reducing Species

H_2_ is the most atom economical reducing agent. The adsorption and activation of H_2_ is a critical step in hydrogenation reaction.^[^ ^1^ ^]^ In thermal hydrogenation, it is difficult to dissociate H_2_ on Au catalyst under equilibrium conditions. The binding energy and sticking coefficient of H_2_ are weak owing to the fully occupied d‐orbitals of Au. Delightedly, this dilemma can be broken in the photocatalytic field. Some researchers reported that photoexcited electrons were transferred to the H_2_ LUMO orbital and thus aided the H_2_ splitting at the metal surface to increase reaction rate.^[^ ^30^ ^]^ Mukherjee et al. reported that hot electrons excited on Au NPs can transfer into Feshbach resonance of H_2_ adsorbed on the surface of Au NPs to trigger H_2_ dissociation under visible light at room temperature. H_2_‐D_2_ exchange experiment was conducted to evaluate the H_2_ dissociation process: An 6‐fold increase of the HD formation rate was achieved under 2.4 W cm^−2^ light illumination.^[^ ^57^ ^]^

##### NaBH_4_ as the Reducing Agent

NaBH_4_ is widely used in the conventional hydrogenation reactions which faced the challenge of low catalytic activity in some cases. Fu and co‐workers^[^ ^28^ ^]^ constructed Au/C_3_N_4_ heterojunction for photocatalytic reduction of nitrophenols in the presence of NaBH_4_. The reaction kinetics on Au@C_3_N_4_ can be greatly accelerated under light irradiation (reaction rate constant, *k* = 7.9895 × 10^−3^ s^−1^) compared to that in the dark (*k* = 5.9362 × 10^−3^ s^−1^). Under visible light illumination, the semiconductor support (C_3_N_4_) can be excited to produce energetic electrons in the conduction band (CB), and the photogenerated electrons transfer rapidly through Schottky barrier to Au nanoparticles which serve as electron reservoir. So the Fermi level of Au shifts to a higher value for the enhanced activation of substrate, which accounts for the higher reaction rate.

When plasmonic metal is attached to the semiconductor, photocatalytic process becomes more complicated due to multi charge transfer routes. Barbosa et al.^[^ ^30^ ^]^ investigated the contribution of light excitation to the hydrogenation of 4‐nitrophenol employing H_2_ or NaBH_4_ as reducing agent over plasmonic Au NPs. When Au is anchored to SiO_2_, the hot electrons on Au NPs can be directly transferred to absorbent, and obvious enhancement in activity was observed for both the two hydrogenation pathways, which is consistent with that on individual Au NPs. In comparison, the positive/negative effect on the plasmonic catalytic activity is reaction‐pathway dependent over Au/TiO_2_ system. In the presence of TiO_2_, the hot electrons are transferred to the CB of TiO_2_ rather than the reactant when NaBH_4_ was used as reducing agent, and the remaining holes on Au NPs will consume part of H^−^ active species, resulting in a decreased activity. However, the preferential transfer of hot electrons to H_2_ LUMO orbital rather than TiO_2_ was observed when H_2_ was used as reducing agent, which is responsible for the promoted conversion. Both of the band structure and the nature of reducing agent determine the transfer direction of charge, which in turn affects the final photocatalytic performance.

#### 2.3.2 Semiconductor‐Based Photocatalytic Systems

The photocatalytic hydrogenation process on semiconductor photocatalyst can be summarized as follows: 1) semiconductor harvests light to induce the separation of photoexcited electrons and holes; 2) the photogenerated electrons transfer to the conduction band (CB) and holes remain in the valence band (VB); 3) the photogenerated electrons at CB can be active toward organic hydrogenation reduction without H_2_ due to the formation of alternative reducing species. There are mainly three types of active species generated in the semiconductor‐based photocatalytic system to trigger reduction process: 1) the photogenerated electrons (Route 3); 2) atomic hydrogen originated from the reduction of hydrogen proton (Route 4) or the dissociation of H_2_ (Route 5), and 3) free radical induced from the oxidation of sacrificial agent by photogenerated holes possessing a higher redox potential than that for the organic reduction (Route 6); these reaction routes provide new schemes for energy saving and green chemical transformation.

##### Reduction by Photogenerated Electrons with H^+^ Assistance

It is worth clarifying that the solely use of MOFs for photocatalytic hydrogenation of organics is still in its infancy. Xing's group^[^ ^18^ ^]^ developed a novel visible‐light‐responsive Zn‐MOF for the photocatalytic reduction of nitrobenzene to aniline. It was found that the reaction induced by direct photoexcited electrons reduction did not occur without hydrogen protons. Once hydrazine hydrate was introduced into the reaction system which acted as hydrogen source and sacrificial agent, nitrobenzene could be reduced to aniline effectively. Furthermore, by introducing a suitable hydrogen source, e.g., H_2_O, alcohol, and formic acid, the reaction also proceeded efficiently. Theoretically, the conduction band potential of a photocatalyst should be lower than the redox potential of reactant for triggering photocatalytic reduction reaction (E(C_6_H_5_NO_2_/C_6_H_5_NH_2_) = −0.486 V, vs normal hydrogen electrode (NHE)). The reduction of nitrobenzene can also be realized by photogenerated electrons derived from the coupled and well‐matched CdS/C_3_N_4_ composite under illumination (ECB = −0.5 V, vs NHE). In this system, the hydrogen atoms were removed from aromatic alcohol assisted by photogenerated holes, and nitrobenzene obtained photoinduced electrons as well as the hydrogen protons. Consequently, aniline was formed.^[^ ^12^ ^]^

##### Both Photogenerated Electrons and Free Radicals as the Reducing Species

Most hole sacrificial agents only provide electrons and hydrogen atoms for the system. However, some hole sacrificial agents can not only suppress the recombination of e^−^ and h^+^, but also produce reducing free radicals via their oxidation reaction on photoexcited holes. Liang group^[^ ^49^ ^]^ demonstrated that CdS@MIL‐68 possessed excellent photocatalytic performance toward the hydrogenation of 4‐nitroaniline (4‐NA) to *p*‐phenylenediamine (PPD). The radicals of ∙CO_2_^−^ were formed in the redox reaction between HCOONH_4_ and the photogenerated holes. It was revealed that the ∙CO_2_^−^ radical is another active species apart from the photogenerated electrons, which was confirmed by electron trapping experiment and ESR study. Both species have enough reductive capability for converting 4‐NA to PPD (E_CB_(CdS) = −1.1V vs NHE; E(∙CO_2_^−^/CO_2_) = −2.2V vs NHE; E(4‐NA/PPD = −0.67V vs NHE).

##### Atomic Hydrogen as the Active Reducing Species

When metal (M) promoter, such as Pt and Pd, is supported on semiconductor, the photoinduced electrons can be efficiently trapped by metal promoter, and thus the hydrogen protons adsorbed on metal surface can be reduced by photoexcited electrons to produce atomic hydrogen. Inspired by these results, Tsutsumi^[^ ^42^ ^]^ and co‐workers exploit another visible‐light‐driven hydrogenation reduction route using Pd‐loaded silicon as the photocatalyst. In this photochemical reduction system, Pd played dual roles during the hydrogenation process. The semiconductor silicon adsorbed photons to generate photoexcited electrons and holes, and then the photoexcited electrons were transferred to Pd nanoparticle and captured by hydrogen protons to generate atomic hydrogen on the Pd surface. Meanwhile, nitrobenzene adsorbed on Pd and reacted with active hydrogen (Pd–H) to form aniline. The adsorption and dissociation of H_2_ molecules can easily occur on noble metal surface, such as Pt, Pd, and Rh, due to its strong interaction with molecular hydrogen.^[^ ^1^ ^]^ The partially occupied d‐orbitals can accept the electron of H_2_, meanwhile the d‐electrons can donate to the δ* antibonding orbital of H_2_, and thus H—H bonds are cleaved and M–H species are formed. Increasing the electron density of metal sites properly will provide enhanced hydrogenation performance, which can be achieved by photocatalytic process. Some researchers loaded Pd NPs on a semiconductor support, like SiC or Cu_2_O, to realize the transfer of photogenerated‐electron from semiconductor to Pd under light excitation, thus the electron density of Pd was increased which further boosted hydrogenation activity.^[^ ^31^, ^34^ ^]^

##### Both Photogenerated Electrons and Hydrogen Atoms as the Reducing Species

In this case, both photogenerated electrons and hydrogen atoms exist in the reaction system. Transition‐metal phosphide is a well‐known catalyst for photocatalytic hydrogen evolution. When CoP was coupled with CdS semiconductor, the obtained heterojunction displayed an unique catalytic process.^[^ ^47^ ^]^ On one hand, CdS adsorbing the energy of photons was excited to produce energetic electrons for the reduction of 4‐nitrotoluene. On the other hand, CoP, acting as a cocatalyst, received photogenerated electrons to generate atomic hydrogen or H_2_. Specially, the atomic hydrogen on CoP can also reduce nitro group, which was confirmed by the atomic hydrogen trapping experiment. Kinetic isotope investigation indicated that the O—H bond cleavage was a critical factor affecting the whole performance.

### 2.4 Photoinduced Selectivity Changes

#### 2.4.1 Selectivity Change Caused by Different Activation Energy Levels of Different Reaction Substrates

**Figure 4:**
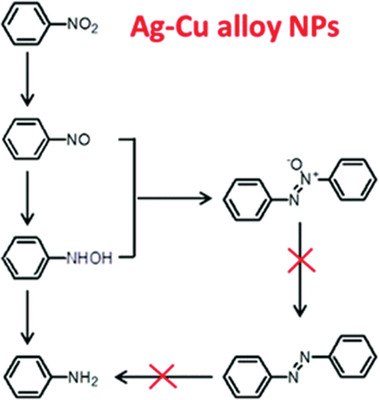
Possible reaction pathways for the reduction of nitrobenzene on Ag–Cu alloy NPs under the irradiation of visible light. Reproduced with permission.^[^ ^56^ ^]^ Copyright 2016, Royal Society of Chemistry.

As illustrated in Section 2.1, photocatalyst can adsorb light and generate excited electrons. The energetic electrons are able to inject into corresponding energy level orbitals to activate the reactant molecules adsorbed on the active sites, which initiates the reduction reaction that is difficult to occur under dark condition. Therefore, adjusting the state of the energetic electrons for selective activation of the substrate can control the degree of hydrogenation reaction. Consequently, the selectivity of the intermediate product will be finely regulated. In other words, acquisition of target intermediate product requires adjusting the energy level of excited electrons to an appropriate energy level which matches with specific reaction. Both Au and Cu metals can strongly adsorb visible light through the localized surface plasmon resonance (LSPR) effect. The energy of their hot electrons depends on the energy of the photon they adsorb. Au and Cu NPs have been used for reductive coupling of nitroaromatics to azo compounds under visible light.^[^ ^58^ ^]^ However, for the hydrogenation of aromatic nitrocompounds, it is challenging to achieve high yield of their corresponding azo compounds. Liu and co‐workers successfully fabricated Ag–Cu alloy NPs to efficiently convert nitrobenzene to azoxybenzene driven by visible light irradiation under mild reaction conditions,^[^ ^56^ ^]^ which can be ascribed to the limited activation capacity of the energetic electrons. This system has the following advantages: 1) the activity can be promoted by increasing the light intensity; 2) the product distribution (azoxybenzene and aniline) can be manipulated by tuning the wavelength of light source. By transferring energetic electrons to nitrobenzene, the formation of aniline needs greater energy than that of azoxybenzene. This work provides a new insight into the photocatalytic system for selective hydrogenation (**Figure** **4**).

#### 2.4.2 Selectivity Change Induced by Electron Density of Active Sites

**Figure 5:**
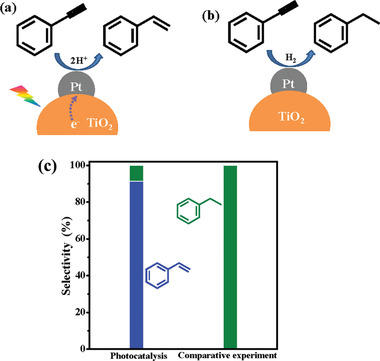
a,b) Illustration of the photocatalytic hydrogenation of phenylacetylene over Pt/TiO_2_ in methanol suspension. c) The comparative experiments with H_2_ as hydrogenation source. Reproduced with permission.^[^ ^43^ ^]^ Copyright 2020, Elsevier.

In thermal catalysis, one of the strategies for selective hydrogenation is to tune the electronic state of the active metal sites. This idea was usually realized by introducing a second metal into the primary active metal. But the increased selectivity often accompanied by a sacrifice of its activity due to the coverage of active surface. In addition, the catalyst suffers from multistep fabrication processes. The regulation of electronic state for selective hydrogenation can be also realized by the photoexcited process. On the easily prepared Mott–Schottky photocatalysts, the electron density of active metal species can be regulated by altering light intensity. An example is the semihydrogenation of triple bond, which is sensitive to electronic density. Lian group^[^ ^43^ ^]^ demonstrated Pt/TiO_2_ photocatalyst exhibit high activity and selectivity for phenylacetylene hydrogenation. Pt/TiO_2_ provided electron‐rich Pt surface with enhanced repulsion of C═C bonds, which favored the desorption of styrene under 385 nm monochromatic light irradiation. Then the selectivity toward styrene was increased (**Figure** **5**).

#### 2.4.3 Selectivity Regulated by the Band Structure of Photocatalyst

Photogenerated electrons may enable the selective reduction of specific functional group owing to its matched redox potential. Therefore, the product selectivity can be regulated more precisely and the yield of by‐products can be effectively suppressed, which can be realized in photocatalytic hydrogenation. Yamamoto et al.^[^ ^39^ ^]^ revealed that DHN/TiO_2_ (DHN is 2,3‐dihydroxynaphthalene) generating photoexcited electrons with suitable reduction potential showed excellent chemoselectivity toward the nitro group while the other reducible groups were remained under H_2_‐free condition. The CB of TiO_2_ (−0.3 V) is more negative than the reduction potential of nitrobenzene (0.16 V), while the half‐wave reduction potential of styrene (−2.41 V) is more negative than CB of TiO_2_. This type of photocatalysts avoids the addition of cocatalyst like noble metal which tends to induce excessive hydrogenation. Besides, the author emphasized that a strong substrate adsorption ability was another requisite to realize the effective transfer of electrons from semiconductor to the substrate.

#### 2.4.4 Selectivity Regulated by the Active H Species

When H_2_ molecules adsorb on Pd, active H atoms (H∙) and Pd–H hydrides can be generated simultaneously. However, the Pd–H always results in over hydrogenation process. Therefore, promoting the formation of H∙ while inhibiting the generation of Pd–H can enhance the selectivity of desired product. Wu's group developed Au–Pd bimetallic nanoparticles supported on SnNb_2_O_6_ ultrathin nanosheets (SN) as the photocatalyst for the selective hydrogenation of cinnamaldehyde to hydrocinnamaldehyde .^[^ ^36^ ^]^ Au_6_–Pd_1_/SN exhibited 99.8% conversion and 91% selectivity under light illumination, while the conversion and selectivity were 23.4% and 52.5% in the dark, respectively. EPR spectra demonstrated that the content of H∙ was significantly enhanced under light irradiation. The trapping of photogenerated electrons by Pd NPs trapped favored the generation of H∙ and suppressed the formation of Pd–H species.

On the whole, light renders several hydrogenation reactions easier to be triggered at mild conditions via the conversion of light input to chemical energy. Meanwhile, the reaction process, following the photocatalytic hydrogenation mechanism, could be proceeded simultaneously through multiple pathways, which remarkably accelerates the activity. More notably, the controllable regulation of selectivity by photocatalysis makes the hydrogenation reaction more tunable and proceed as expected.

## 3 Key Reaction Conditions

In the field of heterogeneous photocatalysis, the fine design of catalyst structure is the primary core of high performance. The reaction conditions, which are closely related to the catalyst structure and reaction type, are also essential in achieving robust catalytic efficiency. In consequence, exploring suitable reaction conditions is a crucial research orientation. Many researchers explored the performance difference of the photocatalyst under different reaction conditions, including light intensity, light wavelength and sacrificial agent, and solvent.^[^ ^33^, ^36^, ^59^ ^]^ In this section, the influences of the critical parameters of light source and sacrificial agent are systematically demonstrated. Besides, the in situ characterizations utilized in photocatalysis are also systematically discussed for an in‐depth understanding of the reaction mechanism.

### 3.1 Light Intensity

The charge state of active site is a critical factor affecting photocatalytic performance, since the “catalyst → substrate” electron transfer is responsible for reactant activation and reduction in electron‐induced reduction reaction. The number of photogenerated electrons is proportional to the activity of the catalyst. Meanwhile, the number of photogenerated electrons increases with the enhancement of light intensity for pristine semiconductor and heterojunctions. If semiconductor contacts with metal, especially plasmonic metal, to form Schottky junction, the charge carrier dynamics become complex. Under strong light illumination, the plasmonic band of metal could be excited to generate energetic hot electrons, which are able to cross Schottky barrier and inject back into the CB of semiconductor as confirmed by ultrafast spectroscopy.^[^ ^60^ ^]^ In other words, the electron state of metal is controlled by the competition between the Schottky junction and the plasmonic effect at different light intensity.^[^ ^61^ ^]^ Besides, the influence of photothermal effect cannot be ignored under high light intensity.

### 3.2 Wavelength of Light

In photocatalytic system, the electrons need to be excited by photons with essential energy (*hv* > energy gap (*E*_g_)) from VB of semiconductor to its CB. For metal nanoparticles, its behavior is intrinsically different from those for semiconductors. The energy of electrons derived from interband transitions and collective oscillation through SPR effect can be tuned by altering the wavelength of light. The shorter the wavelength is, the greater the distribution of electrons with higher energy.^[^ ^54^ ^]^ Thus, the wavelength of employed light source is an important factor impacting efficiency of light adsorption and energy of the excited electrons. Generally speaking, it is effective to adjust the wavelength of light to meet the band gap of employed photocatalyst to maximize the utilization of photons. The action spectrum is an evaluation method: the absorption spectrum of photocatalyst is correlated with its photocatalytic activity to identify whether the reaction depends on the density or the energy of electrons. Wu et al. discovered an enlightening observation: In a reaction that can easily occur in the dark, the reactant is strongly adsorbed on the catalyst, thus the photoexcited electrons with moderate energy can further facilitate the reaction. In such situation, the amount of photogenerated electrons, instead of their energies, is the critical factor affecting photocatalytic activity. Then, the catalyst adsorbing light strongly exhibits superior activity. When the reactant weakly adsorbs on the catalyst, short‐wavelength light illumination with high energy is necessary to initiate the photocatalytic process.^[^ ^62^ ^]^ It is necessary to control the intensity/energy of light used in photocatalysis. Mas‐Ballesté stated that high‐power/energy light can lead to the decomposition of material and the occurrence of undesired side reactions.^[^ ^26c^ ^]^

### 3.3 Sacrificial Agent

The sacrificial agent also greatly affects the behavior of photocatalytic reaction. The compatibility and stability of sacrificial agent under experimental conditions have great influence to photocatalytic outcome. Alcohols are commonly used as sacrificial agent in photocatalytic system, their suitable trapping behavior of holes can greatly reduce the rate of e^−^–h^+^ recombination. Protons, generated from the oxidation of trapping agent, and photogenerated electrons can participate in the reduction reaction. Yang's group investigated the influence of different alcohols on the photocatalytic reduction of nitrobenzene, including methanol, ethanol, isopropyl alcohol and *n*‐butyl alcohol.^[^ ^63^ ^]^ In their system, secondary alcohol outperformed primary alcohol, and isopropyl alcohol exhibited the best activity among these four solvents. There are other sacrificial agents, such as organic acid and amine chemicals, which can be adopted by specific experimental system. For the selection of sacrificial agent, both reaction type and catalyst structure should be considered.

### 3.4 In Situ Characterizations Used in Photocatalysis

In‐depth understanding of photocatalytic mechanism is the perquisite to achieve enhanced performance. Structure changes of the catalyst, reaction barriers, rate‐controlled steps should be clearly explored. In situ characterizations are the effective tools to probe reaction process, monitor catalyst changes, illustrate adsorption behaviors of intermediate species on the catalyst, and determine charge transfer pathways. In return, we can guide multifactors to work in a synergistic way. The most commonly utilized in situ technologies are summarized in this section.

#### 3.4.1 In Situ Irradiated X‐Ray Photoelectron Spectroscopy (XPS)

The migration pathway of photogenerated carriers across photocatalyst can be illustrated by monitoring the binding energy shift of constituent elements before and after light irradiation.^[^ ^64^ ^]^ Yu group fabricated a Z‐scheme TiO_2_/CdS heterojunction for photocatalytic CO_2_ reduction, which was confirmed by in situ XPS. The Ti 2p binding energy displayed positive shift upon light irradiation, while the Cd 3d peak underwent negative shift at the same time. It suggests that the photogenerated electrons migrate from TiO_2_ to CdS, which is in good agreement with the direct Z‐scheme mechanism.^[^ ^65^ ^]^ In situ near ambient pressure XPS (NAP‐XPS) can also be employed to monitor the changes of reactant species and reveal reaction pathway during photocatalytic reaction. Xiong et al. collected the time‐dependent NAP‐XPS spectra for N 1s to probe information of N‐containing intermediates during N_2_ photohydrogenation,^[^ ^66^ ^]^ which provides a direct evidence for the tuned local electronic structure of active sites.

#### 3.4.2 In Situ Fourier Transform Infrared Spectroscopy (FTIR)

In situ FTIR can be employed to monitor the reactant/intermediate species and their interactions with the catalyst during reaction process. Yu and co‐workers^[^ ^67^ ^]^ gained insights into the photocatalytic CO_2_ reduction process through in situ FTIR to detect the CO_2_ conversion intermediates on g‐C_3_N_4_/SnS_2_. When g‐C_3_N_4_/SnS_2_ was exposed to CO_2_ and H_2_O in the dark, monodentate carbonate (m‐CO_3_^2−^), bidentate carbonate (b‐CO_3_^2‐)^ and bicarbonate (HCO_3_^−^) were observed in the FTIR spectra. With the light on, two new peaks, assigned to COO^−^, appeared after light irradiation for 1h, indicating that HCOOH was the intermediate component during the photoreduction of CO_2_. Li et al. investigated the adsorption behaviors of different electron donors (formaldehyde, formic acid and oxalic acid) on TiO_2_ through in situ FTIR to explain distinct photocatalytic activities under different conditions. The adsorption intensities of three electron donors are in the order of oxalic acid > formic acid > formaldehyde. The highest photocatalytic activity was obtained in the system of oxalic acid. Because the strongest interaction between electron donor and the catalyst is conductive to the transfer of photogenerated charge.^[^ ^59b^ ^]^

#### 3.4.3 Operando X‐Ray Adsorption Spectroscopy (XAS)

The structure of several catalysts will inevitably change in the photocatalytic process. Therefore, it is necessary to monitor the dynamic structure and composition under experimental conditions. Then, the real structure‐performance relationship of the catalyst can be established.^[^ ^68^ ^]^ Cu element is widely used in photoreduction of CO_2_. Due to the transient changes of structure and valence state of Cu, it is still controversial which Cu structure is the most active species for photocatalytic CO_2_ reduction. In situ XAS analyses were utilized to track the real‐time evolution of Cu species during photocatalytic CO_2_ reduction by Xiong group.^[^ ^69^ ^]^ The in situ XAS results showed that Cu(II) species were gradually reduced to Cu(I) and ultimately to Cu(0) under light irradiation. The mixture of Cu(I) and Cu(0) was considered to be more efficient for CH_4_ formation.

#### 3.4.4 In Situ Electron Paramagnetic Resonance (EPR)

In the photohydrogenation system, multiple active reducing species often exist simultaneously to achieve the final conversion. In situ EPR is a strong tool to detect intermediate radicals and uncover real reaction pathway. Wu group realized a rapid photocatalytic hydrogenation of halonitrobenzene to haloaniline using HCOONH_4_ as the hydrogen source. To elucidate the active species, in situ ESR experiment coupled with 5,5‐dimethyl‐1‐pyrroline N‐oxide (DMPO) spin‐trapping was conducted under light irradiation. The apparent signal of DMPO–∙CO_2_^−^ under light illumination suggested the formation of ∙CO_2_^−^ with strong reducibility, which can couple with H^+^ to reduce the –NO_2_ group.^[^ ^50^ ^]^

Overall, the irradiation intensity and wavelength can be varied to tune the number of the photogenerated electrons and their energies. Then, the ultimate catalytic performance can be effectively improved via controlling the electron state of active sites. In addition, the photocatalytic rate also depends on the ability to supply or obtain electrons of sacrificial agent and its adaptability in the system.

## 4 Photohydrogenation by Metal‐Organic Frameworks (MOFs)‐Based Photocatalysts

Compared with traditional porous materials (such as zeolites, clays or mesoporous silica), MOF‐based materials hold novel properties and particular advantages, and can be rationally designed for the use in hydrogenation reactions. 1) MOFs possess multiple and tunable active species (nodes, linkers, and pores). Moreover, various catalytic sites can be encapsulated into MOFs to develop MOFs‐based catalysts for diverse hydrogenation reactions. 2) The tunable pore structure of MOF can efficiently change the diffusion of reactants to the active sites, and then tune the activity and selectivity of the MOFs‐based catalysts.^[^ ^2^ ^]^ 3) The well‐defined structure of MOFs provides great chance to design active sites at an atomic level and to understand the reaction mechanism at the molecular level.^[^ ^70^ ^]^ 4) The high specific surface area of MOFs can increase the concentration of active sites and enhance the adsorption of substrates.

**Scheme 2:**
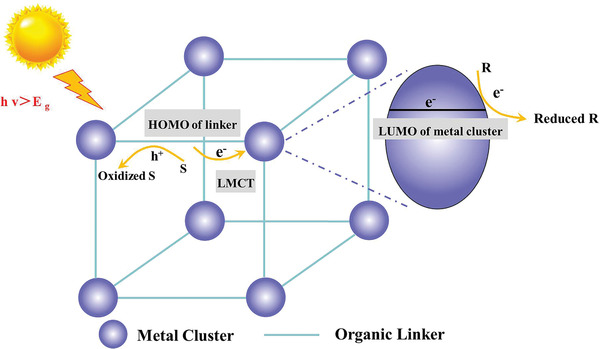
Photoexcitation and carrier migration in MOF semiconductor. HOMO: highest occupied molecular orbital; S: sacrificial agent; R: reactant; LMCT: ligand‐to‐metal charge transfer.

Photocatalytic hydrogenation of organics is considered to be an environment‐friendly process for the production of industrial chemicals as it can directly convert solar energy into chemical energy.^[^ ^71^ ^]^ The unique properties of MOFs make them the ideal alternatives for heterogeneous photohydrogenation.^[^ ^5^, ^72^ ^]^ **Scheme** **2** illustrates the photoexcitation and the charge transfer process in MOFs. Regarding the above analysis on the mechanism of photocatalysis, the main causes of low efficiency for photohydrogenation are limited visible light adsorption, fast photogenerated electron–hole recombination and high reaction barriers.^[^ ^25^, ^73^ ^]^ Many efforts have been devoted to enhance the efficiency for photocatalytic hydrogenation over MOFs‐based catalysts. For instance, through doping metal nodes, functionalizing ligands, loading metal NPs and constructing heterogeneous structures, the light adsorption capacity and charge separation efficiency can be effectively improved.^[^ ^22^, ^25^ ^]^ In the following sections, we will discuss in detail the design of MOFs‐based catalysts to improve the efficiency of photohydrogenation and the recent advances on photohydrogenation of organic chemicals and CO_2_ catalyzed by MOFs‐based composites.

### 4.1 Photohydrogenation of Organic Chemicals

MOFs hold great potential for exploring new prospective catalysts to realize high performance toward photohydrogenation of unsaturated organic substrate. Under light driven, MOFs‐based photocatalysts can result in charge separation to induce direct reduction reaction by photoexcited electrons, which provides a green and new synthesis route for fine products.^[^ ^14b^ ^]^ Moreover, the interconnected channels facilitate the accessibility of high‐density active sites and the transport of substrate/product. Then, the interaction between reactant and photogenerated electrons can be strengthened to increase catalytic activity. Up to date, several MOFs‐based photocatalytic systems have been known to be capable of photoconverting organic chemicals.^[^ ^74^ ^]^ **Table** **2** summarizes the representatively MOFs‐based photocatalysts developed by different strategies for the photocatalytic hydrogenation of organic compounds.

**Table 2:** MOF‐based photocatalysts developed by different strategies for the photocatalytic hydrogenation of organic chemicals

Strategies	Catalysts	Light source	Hydrogen source	Substrate	Active species	Ref.
Modified metal nodes or linkers	Hf/ZrUiO‐66	UV–vis	iPrOH^a^	Nitroaromatic	e^−^	[75]
	Ni‐porphyrin MOF	Visible light	NaBH_4_	Nitroaromatics	BH_4_^−^	[76]
	Zn‐MOF	Visible light	N_2_H_4_	Nitroaromatics	e^−^	[15]
Loading of plasmonic metal NPs	Pd@ZIF‐8	Visible light	H_2_	Olefin	Had^b^	[77]
	Au@Pd@ZIF‐8	Visible light	H_2_	Alkynes	Had ^b^	[78]
	Au/PtAu@UiO‐66‐NH_2_	Visible light	H_2_	Cinnamaldehyde	Had^b^	[78]
Deposition of noble metals	Pt or Au/ NH_2_‐MIL‐101(Fe)	Visible light	HCOOH	Aromatic aldehyde	H^•^	[79]
	Pt@UiO‐66	Full spectrum	H_2_O	Nitrobenzene	Had^b^	[80]
	Pt/Ti‐MOF‐NH_2_	Visible light	H_2_O	Nitrobenzene	H^•^	[81]
Construct heterojunction	Ag/MIL‐125(Ti)/g‐C_3_N_4_	Visible light	iPrOH^a^	Nitrocompounds	e^−^	[82]
	CdS@MIL‐68(Fe)	Visible light	H_2_O	4‐Nitroaniline	e^−^; ∙CO_2_^−^	[49]
	Ce‐doped UiO‐66/graphene	Visible light	iPrOH^a^	Nitroaromatic compounds	e^−^; H^•^	[63]

^a^ iPrOH: isopropanol

^b^ Had: active adsorbed hydrogen species.

#### 4.1.1 Single MOFs Catalysts

MOFs with active sites locating on metal node or organic linker can act as the photocatalyst for some reduction reactions. Nevertheless, most MOFs possess limited light harvest ability and suffer from high photoinduced charge carriers transfer energy. Accordingly, it is necessary to tailor the band structure of MOFs by the design of organic linker with suitable length, geometry, and functional group. Besides, the metal identity of inorganic node also affects MOFs property.

For the reduction of nitrobenzene (NB), the law of reaction thermodynamics should be followed: *E*_CB_ >EC_6_H_5_NO_2_/C_6_H_5_NH_2_ + *E*_R_ (*E*_R_ is the overpotential for nitrobenzene reduction). The more negative potential of CB can provide a stronger driving force for the reduction process. UiO‐66 (Zr_6_O_4_(OH)_4_(BDC)_12_), as a typical MOF, possesses tunable structure, excellent thermal stability and low energy level of CB (−0.6 V, vs NHE at pH 7.0), which is more negative than the reduction potential of NB (−0.486 V vs NHE). Elkin^[^ ^75^ ^]^ compared a series of MOFs in UiO‐66 family with diverse linkers and metals for photocatalytic reduction of nitro‐aromatics, and disclosed the roles of both the metal and the linker on photocatalytic process. The Hf‐based MOF outperformed the Zr analogues. Moreover, the impacts of linker identities on increasing the activity of MOF are in the following order: pyridine (py) > 2‐aminoterephthalic acid (BDC‐NH_2_) > BDC. The enhanced performance of Hf‐py was ascribed to the altered LMCT energy and ability to conduct protons, which is critical to proton‐coupled electron transfer redox reactions. Other groups also reported related research results with respect to the MOF photocatalyst. Jiang et al. showed that NH_2_ can effectively suppress the recombination of photogenerated charge carriers.^[^ ^24a^ ^]^ Li et al. demonstrated that the doping of Ce or Ti into the skeleton of Zr‐based MOFs dictated light adsorption, band structure and LMCT efficiency.^[^ ^23^ ^]^

#### 4.1.2 Coupling MOFs with Carbon Materials

**Figure 6:**
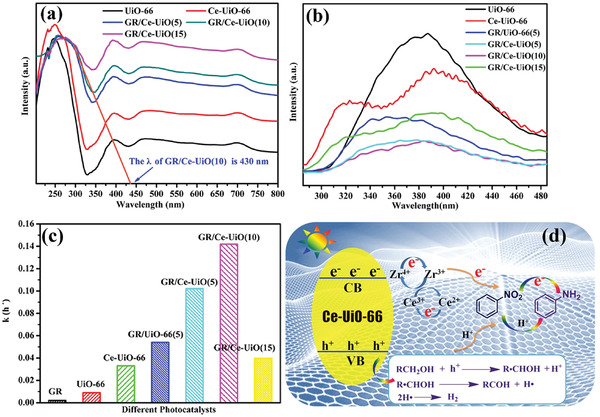
a) UV–vis diffuse reflectance spectra and b) photoluminescence spectra of the samples. c) The rate constant (*k*) of different catalysts for photocatalytic nitrobenzene reduction. d) Possible mechanism for the photocatalytic reduction of nitrobenzene over GR/Ce‐UiO. Reproduced with permission.^[^ ^63^ ^]^ Copyright 2017, Elsevier.

Combing MOFs with carbon materials is another strategy to improve the catalytic performance toward hydrogenation reaction. Previously, multifunctional carbon materials including graphene (GR) and carbon nanotubes (CNT) have been employed as building blocks for photocatalyst carrier/promoter due to their superior conductivity, excellent mobility of charge carriers and abundant active sites. Inspired by these, Yang and co‐workers prepared GR/Ce‐UiO‐66 core–shell hybrids with adequate interfacial contact by a solvent thermal method^[^ ^63^ ^]^ The order of photoreduction rate of nitrobenzene (NB) was as follows: GR/Ce‐UiO66(10) > GR/Ce‐UiO66(5) > GR/Ce‐UiO66(15) > Ce‐UiO66 (the number in bracket refers to the GR amount in the composite). The introduction of appropriate amount of GR can remarkably improve the transfer of photoinduced electron from Ce‐UiO66 and increase the separation efficiency of photogenerated carriers. In especial, GR provides sufficient adsorption sites for NB molecules, which is beneficial to the receiving of photogenerated electrons by NB in the subsequent process to initiate reduction reaction (**Figure** **6**).

#### 4.1.3 Introducing Plasmonic Metal into MOFs

Plasmonic metal nanostructures can be used in photocatalysis due to their strong interactions with electromagnetic radiation through an excitation of localized surface plasmon resonance (LSPR).^[^ ^83^ ^]^ MOF is an ideal candidate for the stabilization of plasmonic metal nanoparticles (MNPs) due to its high porosity and tunable pore size. Incorporating plasmonic NPs into MOFs is a promising way to enhance the catalytic performance of hydrogenation. In the charge‐carrier‐driven reaction, external light is used to excite charge carriers on the metal surface. Few electrons with higher energy can be transferred to the lowest nonoccupied orbit of the adsorbed species, resulting in the activation of chemical bond and chemical transformation. In particular, electron‐driven reaction can potentially target certain reaction pathway, which is unselective in purely thermal reaction. In addition, the photothermal effect converting light into heat has been generated on plasmonic metal with local heating of the lattice, and the reactivity of metal can be promoted by tuning light intensity.^[^ ^61^ ^]^ So far, many researchers reported the successful applications of plasmonic NPs‐MOFs based materials in the field of hydrogenation.^[^ ^84^ ^]^ Two main strategies to improve catalytic performance of MOFs‐based materials by introducing plasmonic NPs are as follows. 1) Immobilizing plasmonic NPs into MOF, tuning their size, geometry, and location for regulating catalytic performance; 2) integrating a second metal together with the plasmonic NPs into MOFs to form an efficient catalyst due to the synergistic effect.

**Figure 7:**
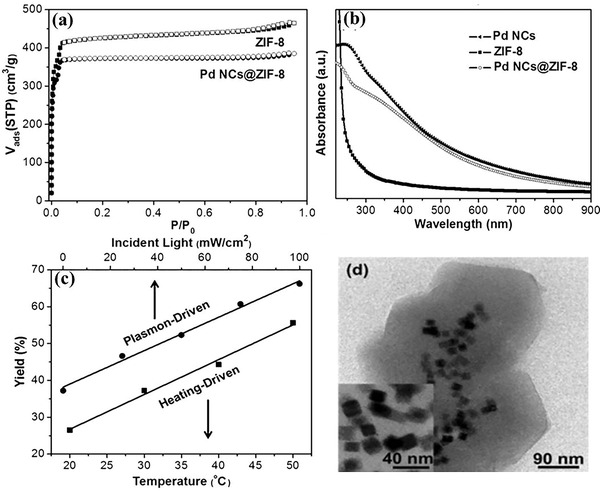
a) H_2_ sorption isotherms for Pd NCs@ZIF‐8 and Pd NCs at 298K; b) UV/vis adsorption spectra for ZIF‐8, Pd NCs and Pd NCs@ZIF‐8; c) the yield of the hydrogenation of 1‐hexene with 1 atm H_2_ over Pd NCs@ZIF‐8 under full‐spectrum irradiation with different light intensities at room temperature or upon heating at different temperatures; d) TEM images of Pd NCs@ZIF‐8. Reproduced with permission.^[^ ^77^ ^]^ Copyright 2016, Wiley‐VCH Verlag GmbH &Co. KGaA, Weinheim.

A plasmonic Pd nanocubes (NCs) @ZIF‐8 composite has been rationally fabricated for selective hydrogenation at room temperature under 1 atm H_2_ and light irradiation.^[^ ^77^ ^]^ The Pd NCs, acting as active sites, have well‐defined structure and maintain high dispersion with the size of 17 ± 3 nm in ZIF‐8. This composite showed a plasmonic band covering the UV‐to‐visible spectral range, which reveals the photon adsorption for inducing high temperature to drive the hydrogenation of olefins. Meanwhile, the ZIF‐8 shell offers the following advantages: 1) the pore structure of ZIF‐8 is beneficial to the transportation of reactant/product and sieving different molecules with specific size for tunable selectivity. 2) It serves as hydrogen reservoir to accelerate the reaction. The reaction efficiency under 100 mW cm^−2^ full spectrum irradiation was much higher than that upon heating at 50 °C, exhibiting the great potential of photothermal effect in the field of catalysis. However, when light is coupled with plasmonic nanomaterial, the hot electron effect and the photothermal effect often work together in the research system, and it is difficult to study a certain type of effect separately (**Figure** **7**). Xiong's group fabricated Au@Pd nanorods, in which the hot electrons excited on Au can directly migrate to Pd, forming electron‐rich Pd surface for the hydrogenation of styrene.^[^ ^78^ ^]^ Both experiments and density functional theory (DFT) calculations revealed the negative effect of hot electrons: the presence of hot electrons did not benefit the hydrogenation reaction due to the strong binding of hydrogen to metal sites. Furthermore, they encapsulated Au@Pd nanorods into ZIF‐8 photocatalytic system for semihydrogenation of alkynes.^[^ ^78^ ^]^ The ZIF‐8 shell hinders the diffusion of heat produced in the plasmonic cores into solution, and maintains a high local temperature for reaction. Thereby, the catalytic activity under light was still 2.5 times that in dark condition even in the presence of mischievous hot electrons. On the basis of the preliminary work, Xiong's group chose Zr‐based MOF UiO‐66‐NH_2_ with larger pore size and higher stability to confine Au NRs‐Pt/Au cores for selective hydrogenation of cinnamaldehyde.^[^ ^78^ ^]^ Thanks to the photothermal effect of Au NRs‐Pt/Au, the reactant conversion reached nearly 100% after 4 h under light, while it was less than 60% in the dark. It is worth noting that cinnamaldehyde preferred to bind to metal sites via the parallel mode under light, which causes the improvement of C═C hydrogenation. Then, the selectivity toward C═O hydrogenation was suppressed. Both the confinement effect of porous UiO‐66‐NH_2_ and electronic structure regulated by light irradiation lead to the higher proportion of C═C hydrogenation product.

#### 4.1.4 Construction Schottky Junction between Noble Metal Nanoparticles and MOFs

**Figure 8:**
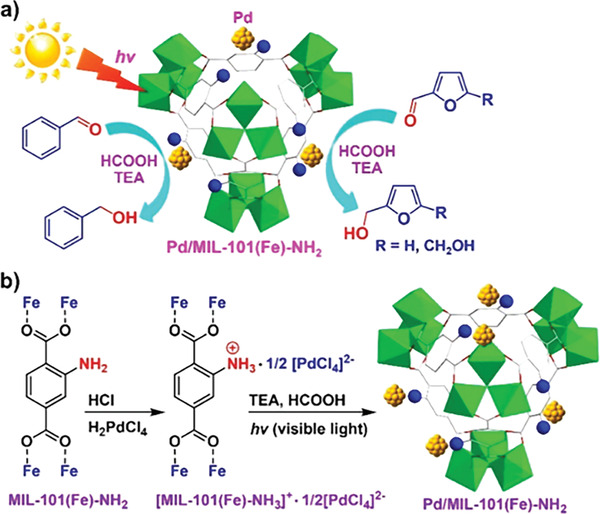
a) Photoinduced transfer hydrogenation of aromatic aldehydes by using Pd/MIL‐101(Fe)‐NH_2_; b) synthesis of Pd/MIL‐101(Fe)‐NH_2_. Reproduced with permission.^[^ ^79^ ^]^ Copyright 2018, American Chemical Society.

Some nanometal‐loaded MOFs are expected to afford high photocatalytic performance of hydrogenation because of the synergic effect between metal NPs and MOFs, in which high distribution of metal NPs, fast molecular transportation, enhanced light utilization and effective photogenerated charge separation are guaranteed.^[^ ^85^ ^]^ The photogenerated electrons can be migrated from MOFs to MNPs which serve as the reservoir of electrons. Rationally loading metal NPs in semiconductor materials had been widely applied to promote the separation efficiency of the photogenerated electrons and holes. Because Schottky barriers can be formed between the semiconductor and metal NPs.^[^ ^86^ ^]^ Moreover, in the terms of the direction of electron migration, there exists a competitive relationship between the hot electron effect and the Schottky junction. In the MNPs/MOF composite, if the energetic electrons excited from MOFs can efficiently transfer to MNPs, the catalytic activity of MNP can be enhanced significantly.^[^ ^34^ ^]^ Inspired by this, Dong and co‐workers^[^ ^79^ ^]^ reported visible‐light‐induced selective transfer hydrogenation of aromatic aldehyde to alcohol catalyzed by Pd/MIL‐101(Fe)‐NH_2_ with triethylamine as electron donor and HCOOH as proton source. The catalyst of Pd/MIL‐101(Fe)‐NH_2_ was prepared by the in situ photodeposition of a Pd salt (MIL‐101(Fe)‐NH_3_)^+^^•^1/2(PdCl_4_)^2−^), possessing highly dispersed and uniform Pd NPs with a mean size around 1.8 nm. Theoretical research confirmed the dual functions of amine group: stabilizing Pd NPs and promoting the electron density of the Pd under light irradiation. Over the Pd/MIL‐101(Fe)‐NH_2_ photocatalyst, the conversion of benzaldehyde was 100% which is much higher than that in the dark (37%). It suggests that the high activity mainly results from the light driving (**Figure** **8**). Ma et al.^[^ ^80^ ^]^ synthesized UiO‐66‐NH_2_ with regulated structural defects, and investigated the effect of these defects on photocatalytic H_2_ production. The H_2_ production rate displayed a volcano‐type trend with incremental levels of defects. Notably, as a preliminary inquiry, the tandem reaction of photocatalytic H_2_ production and nitrobenzene hydrogenation can be promoted without additional hydrogen source, which provides valuable inspiration of engineering MOF‐based photocatalysts for organic hydrogenation transformation.

#### 4.1.5 Coupling MOFswith Semiconductor

**Figure 9:**
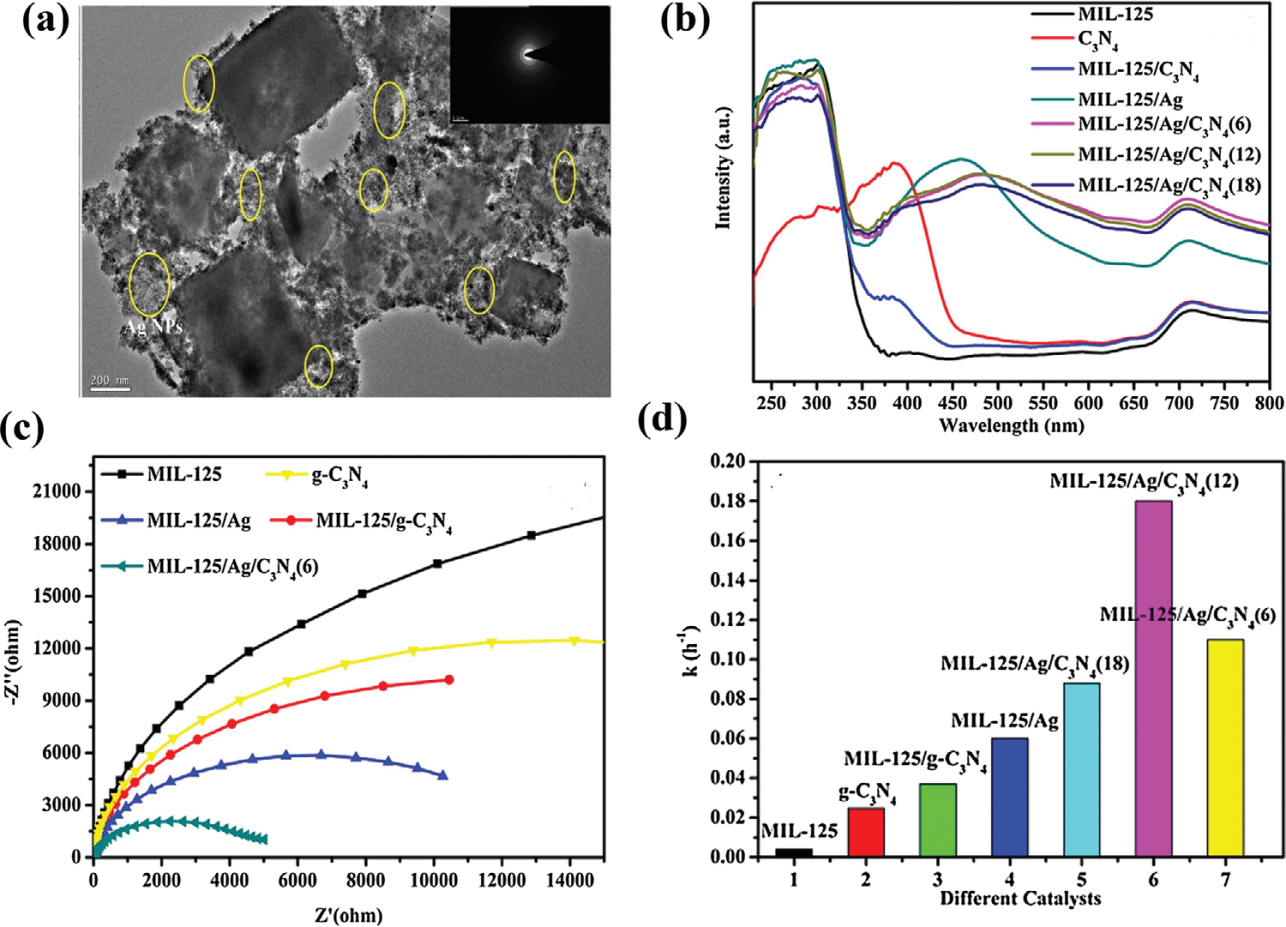
a) TEM images of MIL‐125/Ag/C_3_N_4_(12); b) UV–vis DRS of the samples; c) EIS Nyquist plots of the samples under dark conditions; d) the rate constant (*k*) of different catalysts. Reproduced with permission.^[^ ^82^ ^]^ Copyright 2017, Elsevier.

**Figure 10:**
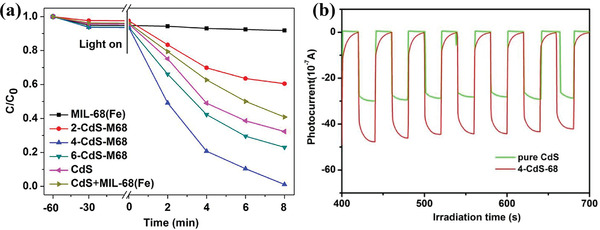
a) Photocatalytic selective reduction of 4‐NA to 4‐PDA over different samples; b) transient photocurrent response of pure CdS and 4‐CdS‐MIL68 in 0.2 m Na_2_SO_4_ aqueous solution under irradiation of visible light (*λ* ≥ 420 nm). Reproduced with permission.^[^ ^49^ ^]^ Copyright 2017, Elsevier.

**Figure 11:**
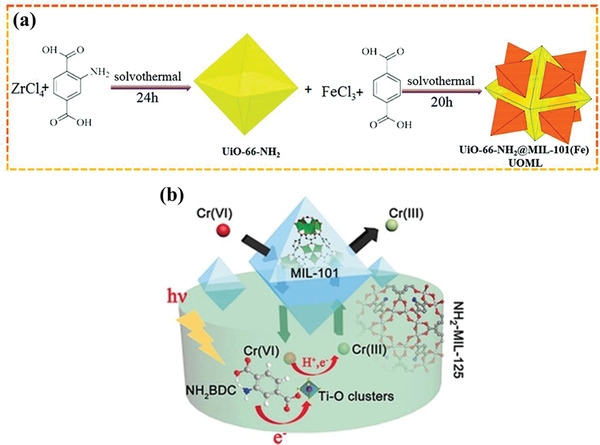
Schematic illustration for the synthetic process of the heterostructures. (a) Reproduced with permission.^[^ ^87^ ^]^ Copyright 2019, Royal Society of Chemistry. (b) Reproduced with permission.^[^ ^88^ ^]^ Copyright 2017, Wiley‐VCH Verlag GmbH &Co. KGaA, Weinheim.

The single‐component semiconductor materials suffer from the low solar energy utilization and the easy photoelectron–hole recombination, resulting in limited photocatalytic performance. Constructing heterojunction by coupling two semiconductors with well‐matched band gap structure is feasible to improve catalytic performance. MOFs is undoubtedly an appreciate alternatives to build heterojunction composite due to its advantages as follows: 1) the facile tuning of band level and light adsorption. 2) highly porous structure with the capability of loading more active sites. A variety of MOFs–semiconductor heterojunctions have been developed and applied in the field of photocatalytic hydrogenation. For instance, a heterostructured bi‐semiconductor material with well‐defined interface was fabricated by coupling Ag (electron‐conduction bridge) and g‐C_3_N_4_/MIL‐125(Ti) for photoreduction nitrocompounds.^[^ ^82^ ^]^ Both g‐C_3_N_4_ and Ag NPs adsorb the visible light, the photogenerated electrons directionally migrate to Ti^4+^ of MIL‐125(Ti) due to the close interfacial connections among MIL‐125(Ti), Ag NPs and g‐C_3_N_4_. Ti^3+^ acted as the active species for the reduction of nitrocompounds due to its strong reducing ability (−1.37V vs SHE). This hydrogenation reduction process avoided using unsafe reduction agent and provided a sustainable and green route for organic reduction. The great potential of MOFs as the photocatalyst for more organic transformation was highlighted. Notably, high selectivity toward various aromatic nitro compounds by this photocatalytic hydrogenation process was observed (**Figure** **9**). CdS has been investigated widely in photocatalytic hydrogenation of 4‐nitroaniline (4‐NA) to *p*‐phenylenediamine (4‐PDA) due to its visible light response and appropriate reductive power. Nevertheless, the low separation efficiency of photogenerated electron–hole pairs and poor stability hinder the application of pure CdS. Liang et al. encapsulated CdS into MIL‐68(Fe) with intimate interfacial contact using a photodeposition method.^[^ ^49^ ^]^ MIL‐68(Fe) not only served as a host to stabilize CdS, but also trapped photogenerated electrons of CdS by forming well‐matched band structure. Trapping experiments and ESR studies revealed that HCO_2_NH_4_ not only served as a hole scavenger but also produced active reducing species (^•^CO_2_^−^). This work made full use of the redox capacity of electrons and holes, providing a highly efficient method for hydrogenation reduction and offering new insight into the design of MOF–semiconductor photocatalyst (**Figure** **10**). Recently, the researches on MOFs@MOFs hybrids have emerged, and these materials showed high potential in the field of photocatalytic organic transformation due to their unique properties superior to their original counterparts (**Figure** **11**). The core–shell UiO‐66‐NH_2_@MIL‐101(Fe) designed by Liu et al. exhibited increased light‐adsorption ability and high charge separation efficiency due to the formation of type II heterojunction with well‐matched band energy.^[^ ^87^ ^]^ Kitagawa et al. reported a MIL‐101(Cr)@MIL‐125‐NH_2_(Ti) heterostructure with improved photocatalytic performance for the reduction of Cr^VI^.^[^ ^88^ ^]^ We believe that Z‐scheme MOF photocatalytic system has considerable potential for photocatalytic hydrogenation and there is still a broad scope for the development in this research area.

### 4.2 Photoreduction of CO_2_ Using MOFs‐Based Photocatalysts

**Scheme 3:**
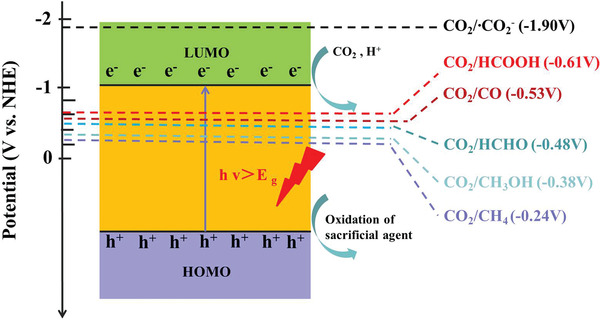
CO_2_ photoreduction process over MOF and redox potential of corresponding products.

At present, a large number of CO_2_ emissions caused a series of environmental problems. Therefore, effectively reducing the content of CO_2_ in the atmosphere, and developing environmentally friendly and renewable new energy sources has become the focus of attention. To this end, a variety of methods for CO_2_ conversion has been developed, such as photocatalytic reduction, electrochemical reduction and biological conversion. Among these technologies, photocatalytic reduction of CO_2_ is considered as a promising technology to obtain chemical or fuel like CH_4_, CH_3_OH, or HCOOH due to its clean and environmentally friendly characteristics.^[^ ^89^ ^]^ In the semiconductor photocatalytic system, photogenerated electrons play the role of reducing active components. Upon light irradiation, the photoexcited electrons were transferred from VB to CB, and subsequently, the photogenerated electrons migrate from CB to catalytic active sites to activate the adsorbed CO_2_ and start reduction reaction. The prerequisite for the reaction to occur is that the CB potential of semiconductor must be negative than the redox potential of CO_2_ reduction for the formation of specific final product (**Scheme** **3**). The final product distribution largely depends on the energy level of CB as well as the reduction potential of the product.^[^ ^90^ ^]^ Due to the high reduction potential of single‐electron reduction of CO_2_ to CO_2_^−^ (−1.90 V), another feasible reduction route is to convert CO_2_ to carbonaceous with the aid of multiple protons and electrons. The reduction potential of CO_2_ into CO, methane, methanol and formic acid are −3.50, −3.79, −3.65, and −3.65 eV, respectively.^[^ ^25b^ ^]^ The activity and selectivity are also affected by other factors, including charge separation efficiency, properties of catalytic center and pore structure.

Based on this background, many MOFs‐based complexes have been demonstrated to be effective photocatalysts^[^ ^91^ ^]^ for CO_2_ conversion, such as ZIF‐8,^[^ ^92^ ^]^ ZIF‐67,^[^ ^93^ ^]^ UiO‐66,^[^ ^94^ ^]^ MOF‐74,^[^ ^95^ ^]^ MIL‐125,,^[^ ^96^ ^]^ HKUST‐1,^[^ ^97^ ^]^ and so on. Their tailored band structure, abundant reaction active sites and superior ability to capture and activate CO_2_ qualify them as excellent catalysts for photoreduction of CO_2_.The activities of those recently developed presentative MOFs‐based catalysts are compared in **Table** **3**.

**Table 3:** Photoreduction CO_2_ activity of recently developed presentative MOFs‐based catalysts

Strategies	MOF	Light source	Reaction condition	Photocatalytic activity	Ref.
Single MOF	MIL‐125(Ti)‐NH_2_	Visible light	MeCN/TEOA	0.814 µmol h^−1^ for HCOOH	[98]
	UiO‐66‐NH_2_	Visible light	MeCN/TEOA	1.32 µmol h^−1^ for HCOOH	[99]
	Ti‐doped NH_2_‐UiO‐66	Solar light	Photovoltaic devices	65.35 µA cm^−2^	[100]
	NH_2_‐MIL‐101(Fe)	Visible light	MeCN/TEOA	22.25 µmol h^−1^ for HCOOH	[101]
	NNU‐31	Visible light	H_2_O	26.3 µmol g^−1^ h^−1^ for HCOOH	[102]
	PCN‐222	Visible light	MeCN/TEOA	3 µmol h^−1^ for HCOOH	[103]
	Zn/PMOF	Visible light	H_2_O	2.6 µmol h^−1^ for CH_4_	[104]
	PCN‐601	Visible light	H_2_O	6.0 µmol g^−1^ h^−1^for CO; 10.1 µmol g^−1^ h^−1^ for CH_4_	[105]
Loading of metal NPs	Au/PPF‐3	Visible light	MECN/C_2_H_5_OH	42.7 µmol g^−1^ h^−1^	[106]
	Ag/MOF‐101(Cr)	Visible light	acetone/TEOA	427.5 µmol g^−1^ h^−1^ for CH_4_; 808.2 µmol g^−1^ h^−1^ for CO	[107]
	Pt/NH_2_‐MIL‐125(Ti)	Visible light	MeCN/TEOA	32.4 µmol g^−1^ h^−1^ for HCOOH	[108]
	Au@UiO‐68‐NHC	Full‐spectrum	MeCN/MeOH	57.6 µmol g^−1^ h^−1^ for CO	[109]
	Au&Pt@ZIF	Visible light	H_2_O	TOF = 1522 h^−1^	[110]
Single atom	Co‐MOF‐525	Visible light	MeCN/TEOA	36.76 µmol g^−1^ h^−1^ for CH_4_	[111]
	Cu/UiO‐66‐NH_2_	Visible light	H_2_O/TEOA	5.33 µmol g^−1^ h^−1^ for CH_3_OH 4.22 µmol g^−1^ h^−1^ for C_2_H_5_OH	[112]
	BUT‐33(Pd)	Full spectrum	H_2_O/TEA	288 µmol g^−1^ h^−1^ for CH4 269 µmol g^−1^ h^−1^ for CO	[113]
Anchoring photosensitizer	UiO‐67(Mn(bpyde)‐(CO)_3_Br)	470 nm	DMF^a^/TEOA	TON 110 for HCOOH	[114]
	MOF‐253 (Rucarbonyl)	Visible light	MeCN/TEOA	1.03 µmol h^−1^ for HCOOH	[115]
	AUBM‐4 (Ru(cptpy)_2_)	Visible light	MeCN/TEOA	366 µmol g^−1^ h^−1^ for HCOOH	[116]
	UiO‐67 (Cp*Rh(bpydc)Cl_2_)	Visible light	MeCN/TEOA	TON 125 for HCOOH	[117]
	Zr‐MBA‐Ru/Re‐MOF	400–800 nm	MeCN/H_2_O	440 µmol g^−1^ h^−1^ for CO	[118]
Constructing heterojunction	TiO_2_@Cu_3_(BTC)_2_	UV	H_2_O	2.64 µmol g_TiO2_^−1^ h^−1^ for CH_4_	[119]
	C_3_N_4_/ZIF‐8	Visible light	H_2_O	0.75 µmol g^−1^ h^−1^ for CH_3_OH	[120]
	UiO‐66‐NH_2_/graphene	Visible light	DMF^a^/TEOA/H_2_O	8.87 µmol g^−1^ h^−1^ for HCOOH	[121]
	Cd_0.2_Zn_0.8_SUiO‐66‐NH_2_	Visible light	H_2_O/Na_2_S/Na_2_SO_3_	6.8 µmol g^−1^ h^−1^ for CH_3_OH	[122]
	Cu_2_O@Cu@UiO‐66‐NH_2_	Visible light	TEOA	8.3 µmol g^−1^ h^−1^ for CH_4_	[123]
	ZnO/rGO/UiO‐66‐NH_2_	Visible light	NaHCO_3_/H_2_O	34.83 µmol g^−1^ h^−1^ for CH_3_OH 6.41 µmol g^−1^ h^−1^ for HCOOH	[124]
	CdS@Co(BDC) MOF	Visible light	MeCN/H_2_O/TEOA	22 µmol g^−1^ h^−1^ for CO	[125]
	ZIF‐67@PPy	Visible light	TEOA/H_2_O/MeCN	1.49 × 10^4^ µmol g^−1^ h^−1^ for CO	[126]

^a^ DMF: *N*,*N*‐dimethylformamide.

#### 4.2.1 Active Sites at the Metal Node

**Figure 12:**
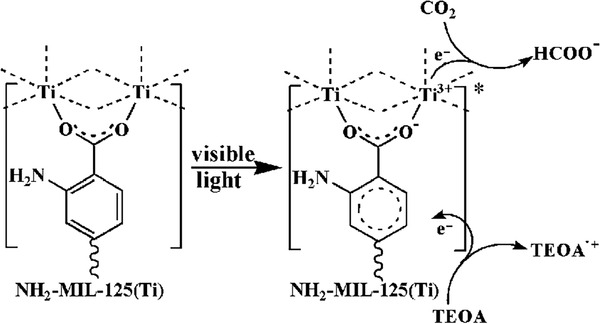
Proposed mechanism for the photocatalytic CO_2_ reduction over NH_2_‐MIL‐125 (Ti) under visible light irradiation. Reproduced with permission.^[^ ^98^ ^]^ Copyright 2012, Wiley‐VCH Verlag GmbH & Co. KGaA, Weinheim.

**Figure 13:**
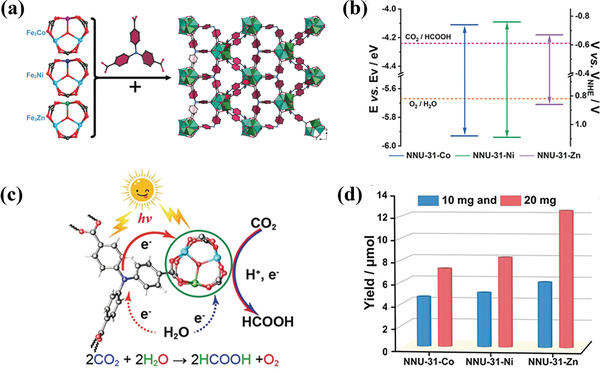
a) 3D framework of NNU‐31‐M constructed by the Fe_2_M cluster and ligand; b) band structure for NNU‐31‐M; c) schematic mechanism of NNU‐31‐M CO_2_RR with H_2_O oxidation; d) the yield of HCOOH on NNU‐31‐M after 24 h. Reproduced with permission.^[^ ^102^ ^]^ Copyright 2020, Wiley‐VCH Verlag GmbH & Co. KGaA, Weinheim.

As early as 2012, Li and co‐workers^[^ ^98^ ^]^ pioneered the application of MOFs to photocatalytic CO_2_ reduction. The photocatalytic reduction of CO_2_ to HCOO^−^ using TEOA as the sacrificial agent under visible light irradiation was realized over NH_2_‐MIL‐125(Ti) containing photoactive Ti. The presence of NH_2_ group not only expanded the optical adsorption but also enhanced the affinity of MOF toward CO_2_. Moreover, they found that Ti^3+^ moiety via photogenerated electron transfer from linkers to Ti^4+^ was responsible for CO_2_ conversion to HCOO^−^ (**Figure** **12**). Compared with Ti^IV^/Ti^III^, Zr^IV^/Zr^III^ has a lower redox potential, which is more conductive to photocatalytic reduction. Based on above considerations, the same group investigated photocatalytic performance of NH_2_‐UiO‐66(Zr) for CO_2_ reduction. As expected, NH_2_‐UiO‐66(Zr) exhibits higher activity for CO_2_ reduction than previously mentioned NH_2_‐UiO‐66(Ti).^[^ ^99^ ^]^ Both studies provided a deep understanding of photocatalytic CO_2_ reduction over MOFs and stimulated researchers to further explore the performance of many other MOFs. The high photocatalytic performance depends on the efficient separation of photogenerated charge carriers. In fact, high‐valence metal ions (e.g., Zr^4+^) and carboxyl groups induce high ligand‐to‐node charge transfer energy barrier, which hinders the reduction reaction. However, the MOFs consisting of low‐valence reactive metal ions are usually suffered from structural lability. Therefore, the doping of metal ions on the metal nodes of MOF is expected as a feasible strategy to promote the separation and mobility of photoexcited charge carriers. Li group further developed a Ti‐substituted NH_2_‐UiO‐66(Zr) via a postsynthetic strategy.^[^ ^100^ ^]^ The role of substituted Ti as the mediator to facilitate electron transfer from excited linker to (Zr/Ti)_6_O_4_(OH)_4_ node to form active sites Zr^3+^ was confirmed by DFT and ESR studies. In addition, direct excitation of metal nodes in MOF also beneficial to photocatalytic CO_2_ conversion. Li and co‐workers^[^ ^101^ ^]^ proposed that amine functionality on Fe‐based MOF can obviously promote the photocatalytic performance for CO_2_ reduction due to the existence of the dual excitation pathways: direct excitations of the Fe‐O clusters and NH_2_ functionalized linkers leaded to the transfer of photogenerated electrons to Fe^3+^ for the generation of Fe^2+^, which is responsible for photocatalytic reduction of CO_2_. Recently, Dong and co‐workers reported that Fe_2_M cluster‐based MOFs NNU‐31 (M = Co, Ni, Zn) converted CO_2_ to HCOOH coupled with the oxidation of H_2_O to O_2_, which is challenging in artificial photosynthesis.^[^ ^102^ ^]^ Unlike ordinary photocatalyst, within this structure, high‐valent Fe^3+^ and low‐valent M^2+^ species acted as oxidation and reduction active sites, respectively, and the 4,4′,4″‐tricarboxytriphenylamine ligand was responsible for the adsorption of light. NNU‐31‐Zn showed the highest HCOOH yield of 26.3 µmol g^−1^ h^−1^ and 100% selectivity for HCOOH (**Figure** **13**).

**Figure 14:**
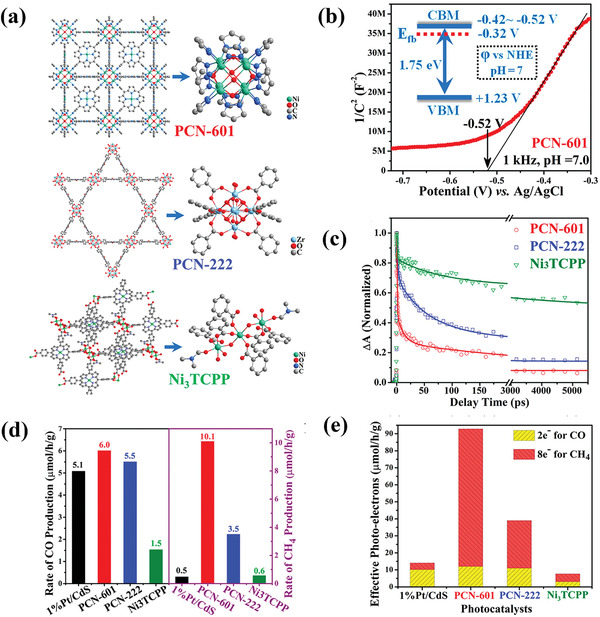
a) Crystal structures and coordination spheres of PCN‐601, PCN‐222, and Ni_3_TCPP; b) Mott–Schottky plot of the as‐prepared PCN‐601 electrode and calculated redox potentials; c) Normalized TA kinetic curves of PCN‐601, PCN‐222, and Ni3TCPP (probe at *λ* = 418nm); d) CO and CH_4_ production rate of PCN‐601 and other reported photocatalysts; e) comparison of the moles of photogenerated electrons utilized in CO_2_ reduction. Reproduced with permission.^[^ ^105^ ^]^ Copyright 2020, American Chemical Society.

Porphyrin is an important class of photosensitive organic compound that adsorbs light in whole visible region strongly. By combining the merits of porphyrin and MOFs, the porphyrin MOFs possess great application potential in photocatalysis.^[^ ^127^ ^]^ The semiconductor‐like PCN‐222, a porphyrinic MOF, has been deliberately employed for effective enriching and converting CO_2_ to HCOO^−^ upon visible light irradiation.^[^ ^103^ ^]^ The ultrafast spectroscopy together with time‐resolved photoluminescence spectroscopy unveil the existence of a deep electron trap state in PCN‐222, which enabled the catalyst to supply long‐lifetime electrons for CO_2_ reduction. Thereby the activity of PCN‐222 was far superior to its H_2_TCPP counterpart. Zhou group rationally developed a pyrazolyl porphyrinic Ni‐MOF (PCN‐601) possessing a larger *π*‐conjugation system for photocatalytic CO_2_ reduction by H_2_O vapor.^[^ ^105^ ^]^ The robust coordination interaction between pyrazolyl and Ni‐oxo nodes endow PCN‐601 with ultrafast ligand‐to‐node electron migration, which is superior to the analogous MOF with carboxylate porphyrin ligand. Accordingly, the photocatalytic activity and selectivity of PCN‐601 for CO_2_‐to‐CH_4_ considerably exceed those of porphyrin‐based MOF and classic Pt/CdS (**Figure** **14**).

#### 4.2.2 Introducing Plasmonic/Noble Metal into MOFs

The composites of plasmonic NPs and MOFs were also developed for photocatalytic CO_2_ reduction.^[^ ^84^ ^]^ Upon visible‐light irradiation, the hot carriers originated from plasmonic metal can be trapped by active sites of semiconductor‐like MOFs to accelerate catalytic process. Chen's group demonstrated that Au NPs acting as light‐harvesting antenna produced hot electrons by SPR excitation, and the energetic hot electrons can be smoothly injected in to Co^2+^, leading to a high density of Co^+^ for selective photocatalytic CO_2_ reduction into HCOOH.^[^ ^106^ ^]^ In this photocatalytic process, the thin support of PPF‐3 nanosheet also contributed to the high yield due to its faster energy transfer and higher mass transport capability. In another work, Guo and co‐workers^[^ ^107^ ^]^ engineered a hybrid catalyst of plasmonic Ag NPs and MOF‐101(Cr) to achieve CO_2_ photocatalytic reduction to CH_4_. In addition to the positive effects of Ag, the tuning of photocatalytic activity by particle size has been examined over nanoscaled MOF. This work provides new ideas for the improvement of photocatalytic performance of MOF materials. Li and co‐workers prepared Pt or Au‐doped NH_2_‐MIL‐125 (Ti), and the effect of noble metal on the photocatalytic performance of CO_2_ hydrogenation was studied.^[^ ^108^ ^]^ Both Pt and Au NPs improved hydrogen production over NH_2_‐MIL‐125 (Ti). However, Pt and Au have converse influences on the photocatalytic performance of formate production. ESR analysis and DFT calculations revealed that dissociated hydrogen can spillover from Pt to the Ti‐O oxo‐clusters, resulting in the formation of active Ti^3+^ sites for CO_2_ reduction. However, the higher energy barrier of hydrogen spillover from Au to the framework of NH_2_‐MIL‐125(Ti) hindered the generation of active sites. This work provides guidance for the development of MNP/MOFs photocatalysts.

**Figure 15:**
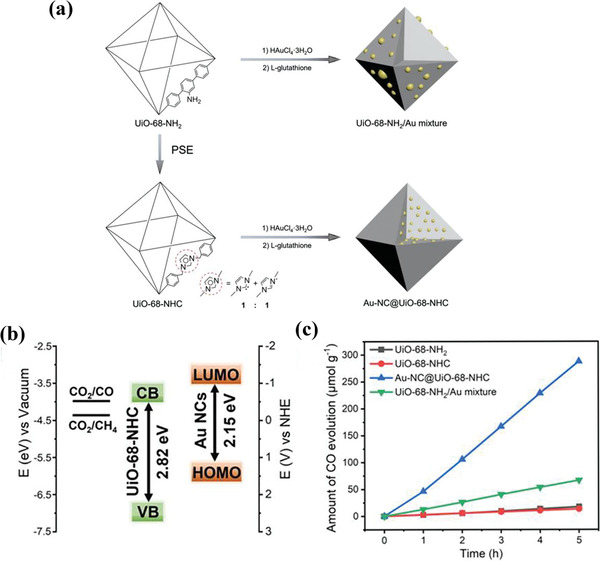
a) Schematic presentation for synthesis of UiO‐68‐NHC, Au‐NC@UiO‐68‐NHC, and UiO‐68‐NH_2_/Au mixture; b) band alignment of Au NCs and UiO‐68‐NHC; c) time courses of CO evolution by photocatalytic CO_2_ reduction using UiO‐68‐NHC, Au‐NC@UiO‐68‐NHC, UiO‐68‐NH_2_, and Au/UiO‐66‐NH_2_ as photocatalysts upon AM 1.5G irradiation. Reproduced with permission.^[^ ^109^ ^]^ Copyright 2021, Wiley‐VCH Verlag GmbH &Co. KGaA, Weinheim.

Construction of tight interfacial contact between MNPs and MOF is a critical step for increasing activity. Jiang et al. reported that the interfacial state played a key role in photoinduced carrier separation. Polyvinylpyrrolidone (PVP), which serves as capping agent to stabilize Pt NPs, presents a negative influence on the interfacial electron transfer between Pt NPs and UiO‐66‐NH_2_. Surface‐clean Pt@UiO‐66‐NH_2_ showed the accelerated sluggish kinetics of electron transfer compared to Pt_PVP_@UiO‐66‐NH_2_.^[^ ^128^ ^]^ Apart from PVP, N‐heterocyclic carbenes (NHCs) were emerged as another stabilization ligand for MNPs via forming stable M‐carbene covalent bonds. In order to load Au NPs into MOF stably, Fei group introduced three stabilization agents into UiO‐68 skeleton respectively, including NHCs, amine and imidazolium.^[^ ^109^ ^]^ In Au‐NCs@UiO‐68‐NHC, the photogenerated electrons can be transferred fast and effectively from Au‐NCs to Zr–O clusters owing to the Au‐NHC covalent bonding. In comparison, the weak noncovalent interactions between amine and Au NPs leaded to limited charge transfer (**Figure** **15**).

Due to the chemical inertness of CO_2_, thermal catalytic hydrogenation of CO_2_ employing H_2_ as reducing agent needs to be carried out at high temperature (200–300 °C) and elevated pressure (50–100 bar). Recently, noble‐metal‐loaded MOFs photocatalysts have attracted great research interest for CO_2_ photothermal catalytic hydrogenation. On one hand, plasmonic metal, like Au NPs, could raise the local temperature by photothermal effect, which increases the activity for CO_2_ hydrogenation. On the other hand, noble metals have superb ability to activate CO_2_ and H_2_ molecules to reduce reaction energy barrier. Zeng's group applied Au&Pt@ZIF photocatalyst to photothermal catalytic CO_2_ hydrogenation.^[^ ^110^ ^]^ Pt nanocubes acted as active sites, and Au nanocages were responsible for converting light into thermal energy to elevate local temperature around Pt sites. Thereby, the TOF value reached to 1522 h^−1^ under light illumination at 150 °C.

#### 4.2.3 Single‐Metal‐Atom MOFs‐Based Photocatalysts

**Figure 16:**
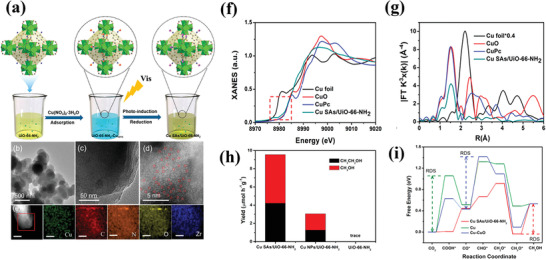
Synthesis process of the Cu SACs/UiO‐66‐NH_2_ photocatalyst; b) TEM and c) HRTEM images of Cu SACs/UiO‐66‐NH_2_; d) aberration‐corrected STEM images of Cu SACs/UiO‐66‐NH_2_; e) EDS mapping of Cu SACs/UiO‐66‐NH_2_; f) normalized XANES; g) EXAFS results of the samples at the Cu–K‐edge; h) comparative rates of CH_3_OH and CH_3_CH_2_OH formation; i) calculated free energy diagram of all steps for CO_2_ reduction to different intermediates on the samples. Reproduced with permission.^[^ ^112^ ^]^ Copyright 2020, American Chemical Society.

Single‐atom catalysts (SACs) with isolated active sites and unique electronic properties, have drawn tremendous attention in the application of thermal catalysis, electrocatalysis and photocatalysis. Theoretically, the maximum atom efficiency and abundant active sites endow SACs with outstanding abilities for effectively separated photogenerated charge carriers and remarkably enhanced activity in photocatalysis. Although great progress has been achieved, the employment of SACs in photocatalytic hydrogenation reactions has been relatively less explored. Ye and co‐workers^[^ ^111^ ^]^ constructed Zr‐porphyrinic MOF (MOF‐525) with Zr_6_ clusters, the catalytically active Co single atoms were introduced by coordinating with porphyrin. The atomic dispersion of active sites greatly boosted the electron–hole separation efficiency in porphyrin units. The electron transfer route can be changed by the introduction of Co center. When the porphyrin center was empty, the photoexcited electrons transfer from the linker to Zr^4+^ for the generation of Zr^3+^. When Co was centered in the porphyrin structure, the photoexcited electrons of the linker will be captured by Co instead of transferred to Zr^4+^. ESR spectroscopy demonstrated the Zr^III^ species disappeared under light irradiation once Co was incorporated into MOF‐525. Co^II^ trapped photogenerated electron excited from porphyrin, and supplied long‐lived electrons for CO_2_ reduction. Meanwhile, the strong CO_2_ adsorption ability also contribute to superior catalytic performance. In 2020, Jiang et al. innovatively reported the regulated photocatalytic CO_2_ reduction by spin‐state manipulation of covalent organic framework COF‐367 featuring Co centered in porphyrin.^[^ ^129^ ^]^ Compared to COF‐367‐Co^II^ (Co^II^, S = 1/2), COF‐367‐Co^III^ (Co^III^, S = 0) exhibited improved activity and significantly enhanced selectivity to HCOOH due to higher photoinduced charge separation efficiency and lower energy barrier for HCOOH formation but higher energy barrier for the further conversion of HCOOH. Li et al. investigated Cu SACs anchored on UiO‐66‐NH_2_ for the photocatalytic hydrogenation of CO_2_ to liquid fuels.^[^ ^112^ ^]^ Notably, only CH_3_OH and CH_3_CH_2_OH were detected in the product. The formation rates of CH_3_OH and CH_3_CH_2_OH for Cu SACs/UiO‐66‐NH_2_ were 5.33 and 4.22 µmol h^−1^ g^−1^, respectively. By contrast, the Cu NPs/UiO‐66‐NH_2_ and UiO‐66‐NH_2_ afforded trace yields under the same condition. Fine characterizations demonstrated the prolonged lifetime of photoinduced charge carriers and enhanced electron capture ability of Cu SACs/UiO‐66‐NH_2_. The introduction of Cu SACs enriched the electrons in Cu SACs/UiO‐66‐NH_2_, which promotes the multielectronic reduction process of CO_2_. Theoretical calculations implied that Cu SACs is a benefit for the formation of CO* and CHO*, leading to highly selective photocatalytic reduction of CO_2_ (**Figure** **16**).

#### 4.2.4 Encapsulation of Catalytic Active Sites

Molecular catalyst possesses high activity toward CO_2_ reduction, but its poor stability makes it impossible to be recycled under reaction condition. Thus, immobilizing molecular catalyst on a support is a good strategy toward the enhancement of catalytic performance. Due to the tenability of pore space and tunability of structures, suitable catalytic active centers can be encapsulated into the framework of MOF. The resulting guest@MOFs may exhibit multifunctional properties owing to the synergistic effect. Cohen and co‐workers^[^ ^114^ ^]^ incorporated molecular Mn(bpyde)‐(CO)_3_Br into UiO‐67 platform, the overall TON for photocatalytic CO_2_ reduction to formate on this Mn‐incorporated MOF reached 110 over 18 h, extensively exceeding to those on bare UiO‐67 and the homogeneous reference system. The increased activity of this catalyst is ascribed to high CO_2_ adsorption capacity, isolated catalytic sites and enhanced stability by Mn‐MOF construction.

**Figure 17:**
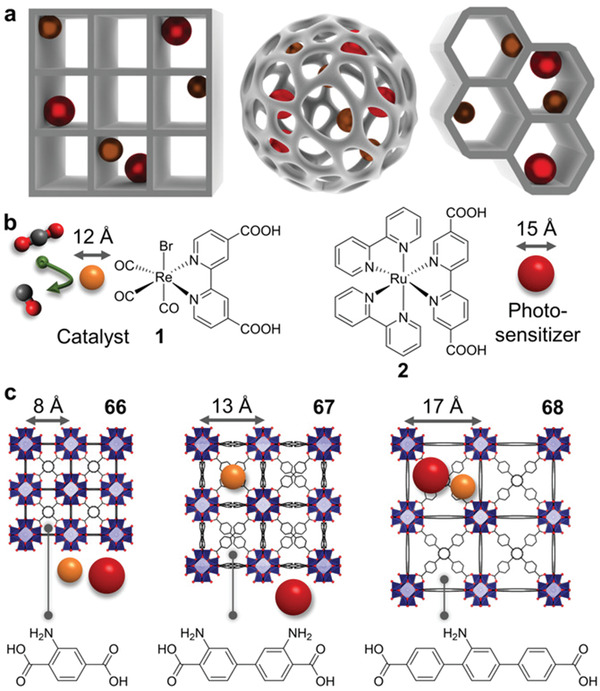
a) Representation of integrated molecular photosystems (spheres) in various assembly controlling MOF topologies. b) Structures of CO_2_ reduction catalyst (ReBr(CO)_3_(4,4′‐dcbpy)) (1) and photosensitizer (Ru(bpy)_2_(5,5′‐dcbpy))Cl_2_ (2). c) Anchoring sites of 1 and 2 in the isoreticular UiO (66, 67, and 68) host series based on pore sizes and the respective MOF linkers. Reproduced with permission.^[^ ^130^ ^]^ Copyright 2021, Wiley‐VCH Verlag GmbH &Co. KGaA, Weinheim.

Sun et al. immobilized active Ru carbonyl complex into MOF‐253 by the formation of *N*,*N*′‐chelating centers for photocatalytic CO_2_ reduction to HCOOH under visible light irradiation.^[^ ^115^ ^]^ In this system, the amount of produced HCOO^−^ was increased by about 12 times compared to the unsensitized one. The significantly improved photocatalytic performance of sensitized MOF‐523 originated from the enhanced visible‐light adsorption and photogenerated electrons injected from ligand to the active sites of MOF‐523. Hmadeh and co‐workers also incorporated photoactive ligand into MOF using a one‐pot method.^[^ ^116^ ^]^ This robust construction, with close proximity between photosensitizing linkers and active centers, enabled the vast charge transfer from ligand to metal, and eventually to the adsorbed CO_2_. Together with its strong adsorption of light, outstanding performance was achieved for CO_2_ reduction. Recently, Stanley et al. immobilized CO_2_ reduction catalyst (ReBr(CO)_3_(4,4′‐dcbpy)) and photosensitizer (Ru‐(bpy)_2_(5,5′‐dcbpy))Cl_2_ onto isoreticular series of UiO‐66, UiO‐67, and UiO‐68.^[^ ^130^ ^]^ The pore size determines the anchoring site (inside vs outside), which has distinct impact on electron communication between reaction center and photosensitizer. This work provided a rational understanding of host‐guest effects, specific anchoring sites and reactive center distance in photocatalytic CO_2_ reduction (**Figure** **17**).

#### 4.2.5 Coupling MOFs with Semiconductor and Carbon Material

TiO_2_ is one of the most widely investigated semiconductor for photocatalysis, which can be applied to construct a heterojunction with MOFs material. Li group^[^ ^119^ ^]^ developed an excellent Cu_3_(BTC)_2_@TiO_2_ hybrid core–shell structure for the conversion of CO_2_ into CH_4_ with the aid of H_2_O. The as‐designed heterojunction material held unique advantages: 1) the photogenerated electrons are effectively transferred from TiO_2_ to Cu_3_(BTC)_2_, which facilitates charge separation and supplies energetic electrons to active CO_2_ molecule adsorbed on Cu sites. The high electron density dramatically improved both activity and CH_4_ selectivity. 2) CO_2_ can be easily captured, passed through the macroporous TiO_2_ coating and adsorbed on Cu sites.

Carbon nitride (C_3_N_4_) is another semiconductor material with visible light activity, low cost and high stability. Liu et al. presented a hybrid composite C_3_N_4_@ZIF‐8 with enhanced CO_2_ adsorption and good optical property. Its photocatalytic efficiency for CH_3_OH production was improved more than 3 times because of the cooperative effect.^[^ ^120^ ^]^

In addition to the binary heterojunction structure, the ternary heterostructure was featured with extra pathways for photogenerated charge transfer to increase photocatalytic efficiency.^[^ ^131^ ^]^ Wang et al. first designed unique ternary Cu_2_O@Cu@UiO‐66‐NH_2_, in which Cu_2_O was coated by UiO‐66‐NH_2_ via a solvothermal method and robust p‐n junction and Schottky barrier were formed.^[^ ^123^ ^]^ Both Cu_2_O and UiO‐66‐NH_2_ could effectively utilize visible light, Cu metal played the role of electron mediator for rapidly delivery of photoexcited electrons from Cu_2_O to UiO‐66‐NH_2_. Consequently, more energetic charge carriers could participate in CO_2_ reduction on the surface of photocatalyst. The reduction of CO_2_ occurred on UiO‐66‐NH_2_, of which the CB potential is above the E(CO_2_/CO) and E(CO_2_/CH_4_). Cu_2_O alone had no activity toward CO_2_ reduction due to the quickly recombination of photoexcited charge electron–hole pairs. Pure UiO‐66‐NH_2_ only produced CO with a formation rate of 6.1 µmol g^−1^ h^−1^. In sharp contrast, Cu_2_O@Cu@UiO‐66‐NH_2_ possessed not only the improved photocatalytic activity but also the promoted CH_4_ formation. Benefiting from this ternary structure, CO can be further converted to CH_4_, which was ascribed to multiple electrons reduction coupled with protons. The yield of CO and CH_4_ reached to 20.9 and 8.3 µmol g^−1^ h^−1^, respectively.

**Figure 18:**
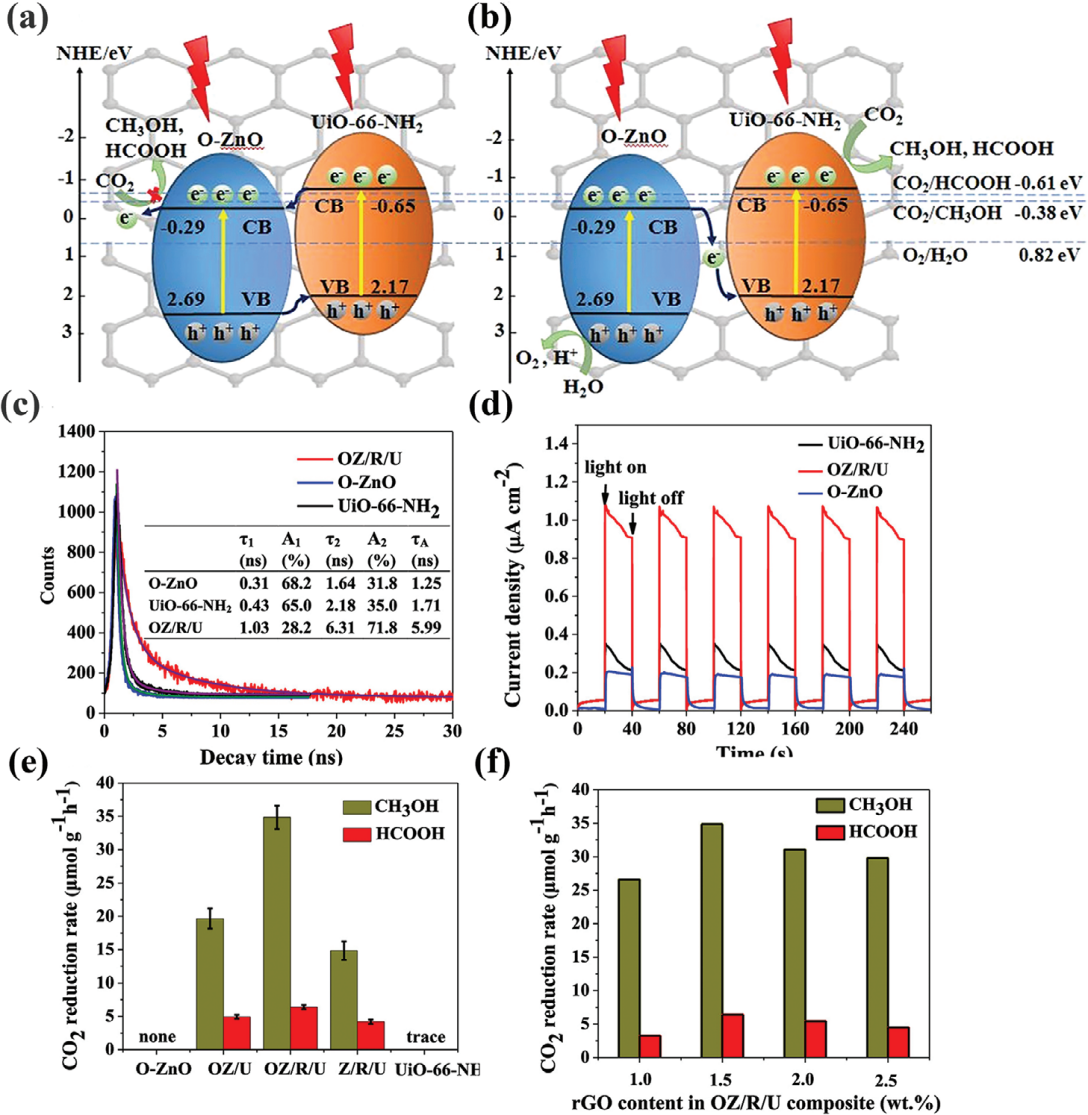
Schematic illustration of the charge transfer and separation in O‐ZnO/rGO/UiO‐66‐NH_2_: a) type‐II heterojunction and b) Z‐scheme mechanism; c) the time‐resolved transient PL decay; d) photocurrent responses; e) CO_2_ reduction rate of the as‐prepared samples; f) effect of the rGO content on the photoactivity of the OZ/R/U photocatalyst. Reproduced with permission.^[^ ^124^ ^]^ Copyright 2019, American Chemical Society.

Most heterostructures are type II heterojunction, which accelerate charge separation by sacrificing their strong redox abilities. These structures are unfavorable for CO_2_ photoreduction from a thermodynamic viewpoint. Satisfactory photocatalytic system for CO_2_ reduction should possess strong light‐harvesting ability, sufficient reduction potential, ultrafast charge separation and good stability. Constructing Z‐scheme photocatalysts is capable to meet above criteria. In 2019, related work on the Z‐scheme heterojunctions based on MOFs (ZnO/rGO/UiO‐66‐NH_2_) was reported by Liu's group.^[^ ^124^ ^]^ The photocatalytic conversion rates of CO_2_ to CH_3_OH and HCOOH on this composite reached 34.83 and 6.41 µmol g^−1^ h^−1^, respectively. These values are much higher than those catalyzed by ZnO/UiO‐66‐NH_2_. Essentially, rGO substrate not only strengthened interfacial contact between O–ZnO and UiO‐66‐NH_2_, but also provided another electron mobility route, which contribute to the dramatically enhanced photocatalytic activity. The recombination of electron–hole pairs of ZnO/rGO/UiO‐66‐NH_2_ was significantly hindered as confirmed by its prolonged PL decay lifetime. Overall, this work gives a new insight into the design of efficient MOF‐based Z‐scheme photocatalytic system (**Figure** **18**).

Overall, MOFs‐based materials are powerful building blocks to construct new photocatalysts toward future prospects. The development of advanced characterizations to gain deep insight of photocatalytic process brings light on reaction mechanism. More and more researchers have devoted their time to explore effective modification strategies of MOFs and their composites for the photocatalytic organic transformation. With the in‐depth understanding of the relationship between catalyst structure and specific photocatalytic process, MOF‐based composites can be rationally designed. These tunable MOFs‐based catalysts will definitely be promising candidates for high‐performance photohydrogenation reactions.

## 5 Summary and Perspective

In this review, we illustrate photocatalytic hydrogenation mechanism and summarize recent developments in MOFs‐based photocatalysts for hydrogenation reactions. Sufficient light adsorption capacity, fast photoexcited charge carriers migration and long lifetime are critical factors affecting photocatalytic efficiency. In addition, the adsorption strength of reactant on active sites has an important influence on the photocatalytic outcome. Proper adsorption of reactant is able to trap electrons rapidly from photocatalysts and thus activate substrate as well as lower the energy barrier of hydrogenation. More trapped electrons and longer lifetime are necessary prerequisites for multielectrons participated reduction reaction. Weak affinity of reactant molecule on the catalyst is less likely to receive photoexcited electrons efficiently, then the effect of light illumination is negligible.

MOFs‐based photocatalysis as a newly promising environmentally friendly technology has been employed in organic synthesis. However, considering the complexity of photocatalytic hydrogenation processes and catalyst structure diversity, it seems that this field is still in its infancy. It is necessary to fully consider whether light irradiation can be a promoter for hydrogenation reactions. As mentioned above, the injection of hot electrons into reactants is favorable for initiating or accelerating chemical reaction, but this is not always the case. For instance, the extra hot electron on Pd surface make a detrimental effect for the dissociation of H_2_,^[^ ^132^ ^]^ which is undoubtedly suppress the hydrogenation process. In fact, the catalytic activity is partially ascribed to substrate adsorption energy of the catalyst. High adsorption strength induced by electron injection is detrimental to catalytic activity. In addition, the effect of LSPR on hydrogenation reaction is closely related to the reaction pathway, which should be taken into account for the design of plasmonic photocatalysts. In the case of CO_2_ reduction, the selectivity of photoreduction products is difficult to be regulated by the CB of photocatalyst, the final product distribution is closely related to the structure of the catalyst. So it becomes imperative to investigate the reaction mechanism deeply and fully understand the effects of key factors on hydrogenation reactions prior to material structure construction and specific use of irradiation condition.

At present, in most cases, the photocatalytic hydrogenation reactions involve only reductive routes with the aid of hole scavengers as electron acceptors, resulting in inevitably increased cost, limited economic added value and formation of oxidized byproducts.^[^ ^26b^ ^]^ Recently, researchers have focused on dual‐functional photocatalytic reaction systems coupling organic reduction and oxidation reactions. Efficient utilization of both photogenerated electrons and holes for the production of high value‐added organic chemicals can be achieved in this reaction system. Dai's group has constructed a coupled system for the reduction of nitrobenzene to aniline and the selective oxidation of aromatic alcohols to aldehydes.^[^ ^26b^ ^]^ H_2_ is considered as a green and renewable energy source.^[^ ^22^ ^]^ Photocatalytic hydrogen generation has been significantly developed in recent year.^[^ ^133^ ^]^ Coupling H_2_ production with organic transformation is an efficient energy utilization pathway and can be realized in photocatalytic systems. Some pioneer researchers combined the hydrogen production process of photocatalyzed water reduction and the hydrogenation process to realize in situ water‐donating transfer hydrogenation.^[^ ^80^, ^81^ ^]^ This solar‐driven eco‐friendly synthesis process has great potential toward a wide range of chemical hydrogenations in the future.

A comparable number of works have employed MOFs‐based catalysts for photocatalytic organic transformation applications. However, the study of photocatalytic hydrogenation application for MOFs‐based materials are still limited. MOFs‐based photocatalysts need to be further improved as useful and stable catalytic system for widely practical applications. Currently, most researches focused on the hydrogenation of simple substrates, such as nitrobenzene. In the future, more hydrogenation processes with more challenging substrate and more complex reaction pathways should be explored. The development of efficient photocatalytic systems significantly relies on the design and fabrication of new materials. MOFs provide great opportunities as a multifunctional platform for the design of active sites and their surrounding chemical environment at an atomic level. On the well‐defined MOFs‐based photocatalyst, the underlying mechanism of photocatalytic hydrogenation can be explored.

1) The light adsorption properties of MOFs can be regulated by engineering ligand structure and the metal ions due to the composition diversity of MOFs. Most of metal nodes, such as Zr–O, Ti–O, and Cu–O clusters, do not have visible light response capability. Thus visible‐light‐responsive metal substitution or doping is a feasible strategy to endow MOFs with superior light harvest ability in the visible light region. Ligand functionalization and photosensitizer introduction are also effective ways. Meanwhile, it is required to finely adjust the band structure of MOFs to meet the reduction potential toward desired reaction. For an efficient MOFs‐based photocatalyst, the lifetime of the photoexcited carriers should be sufficiently long to ensure that the reaction can compete with the decay of the charge separated state.
2) Most MOFs structure features microporous, which makes the tunable selectivity and limited reactivity by the diffusion of substrate. Development of hierarchically porous MOFs is able to reduce mass transfer resistance, and the conversion of the reactants with relatively large size is expected to be realized. However, we should keep in mind that porous structure modification may cause the formation of defective sites, and excessive defects will reduce charge carriers separation efficiency. It is also encouraged to fabricate 2D MOFs nanosheets with abundant available active sites for photocatalytic hydrogenation.^[^ ^134^ ^]^
3) Single‐atom MOFs‐based structures have been employed in the field of thermal catalytic hydrogenation and photoreduction of CO_2_. While these novel materials have not been employed in photohydrogenation of organic chemicals yet. More frontier explorations are needed for this subject in the future. It is worthwhile to develop advanced characterization techniques coupled with theoretical guidance to determine the critical reaction steps, reducing species and exact active sites at an atom level.

In conclusion, this review hopes to provide overall comprehension of photocatalytic hydrogenation and corresponding design idea of MOFs‐based photocatalysts for various organic hydrogenation conversion. The state of the art of photocatalytic applications of MOFs‐based composites for hydrogenation conversion envision a bright future for catalytic reactions utilizing visible light irradiation.

## Conflict of Interest

The authors declare no conflict of interest.
